# Distribution of centrality measures on undirected random networks via the cavity method

**DOI:** 10.1073/pnas.2403682121

**Published:** 2024-09-25

**Authors:** Silvia Bartolucci, Fabio Caccioli, Francesco Caravelli, Pierpaolo Vivo

**Affiliations:** ^a^Department of Computer Science, University College London, London WC1E 6EA, United Kingdom; ^b^Centre for Financial Technology, Imperial College Business School, South Kensington, London SW7 2AZ, United Kingdom; ^c^London Mathematical Laboratory, London WC 8RH, United Kingdom; ^d^London School of Economics and Political Science, Systemic Risk Centre, London WC2A 2AE, United Kingdom; ^e^Theoretical Division (T-4), Los Alamos National Laboratory, Los Alamos, NM 87545; ^f^Department of Mathematics, King’s College London, London WC2R 2LS, United Kingdom

**Keywords:** networks, cavity method, centrality

## Abstract

The Katz centrality of a node in a complex network is a measure of the node’s importance as far as the flow of information across the network is concerned. For ensembles of locally tree-like undirected random graphs, this observable is a random variable. Its full probability distribution is of interest but difficult to handle analytically because of its “global” character and its definition in terms of a matrix inverse. Leveraging a fast Gaussian Belief Propagation-Cavity algorithm to solve linear systems on tree-like structures, we show that i) the Katz centrality of a single instance can be computed recursively in a very fast way, and ii) the probability P(K) that a random node in the ensemble of undirected random graphs has centrality K satisfies a set of recursive distributional equations, which can be analytically characterized and efficiently solved using a population dynamics algorithm. We test our solution on ensembles of Erdős-Rényi and Scale Free networks in the locally tree-like regime, with excellent agreement. The analytical distribution of centrality for the configuration model conditioned on the degree of each node can be employed as a benchmark to identify nodes of empirical networks with over- and underexpressed centrality relative to a null baseline. We also provide an approximate formula based on a rank-1 projection that works well if the network is not too sparse, and we argue that an extension of our method could be efficiently extended to tackle analytical distributions of other centrality measures such as PageRank for directed networks in a transparent and user-friendly way.

## Significance

Centrality measures allow to identify important nodes in networked systems. An open question in network theory is the empirical observation that a node’s centrality—whose computation requires knowledge of the entire network—strongly correlates with its degree (the number of its neighbors), a local observable. We address this puzzle providing an analytical derivation of the distribution of Katz centralities in random networks. Our results explain the connection between degree and centrality: For sparse networks, the distribution of centralities is a multimodal distribution where different peaks correspond to different degrees. This finding suggests that the functionality of empirical networks may be related to nodes with over- or underexpressed centrality. Our results provide a methodology for the efficient identification of such nodes.

## 1. Introduction

The study of complex systems and the applications of the “science of complexity” to the most diverse areas of research have witnessed spectacular successes in recent years. Complex systems are quintessentially defined as being composed of many components that are interacting locally, exhibiting emerging static and dynamical properties, and involving a certain degree of randomness. However, not every elementary constituent plays the same role in the structure or functionality of a system, with some constituents being more critical and “central” to ensure stability, resilience, or other desired global properties of the architecture (1–14). Identifying the most important nodes in a network architecture is indeed of paramount importance to ensure the integrity and functionality of transportation networks and critical infrastructures (15–19), as well as to allow users to retrieve an accurate list of webpages corresponding to an Internet query (20, 21), or identify the most suitable receivers of a vaccine to mitigate a disease outbreak (22–24). Our ability to exploit the advantages of living in a modern and interconnected society to the full heavily relies on preserving the integrity of crucial infrastructures such as the Internet and power grids (1, 25–29).

Several “centrality” measures have been developed to classify and rank nodes of a network, which focus on different structural characteristics: The degree centrality simply counts how many neighbors each node has and ranks nodes according to how locally connected they are. More global centrality measures include the eigenvector centrality (30), the Katz centrality mainly considered here (31), and Google PageRank (20, 21). Other definitions take into account the relative position of each node in the network (for instance, closeness and betweenness (32, 33), communicability (34) and DomiRank (35)), as well as the role played by a node in a dynamic process on networks (for instance, current-flow (36), entanglement (37), and random-walk (38) centralities)—see ref. 39 and references therein for a taxonomy of centrality measures on networks and refs. 40–42 for comprehensive reviews.

When the underlying structure is a single instance or an ensemble of random networks, generated according to probabilistic rules, each of the above centrality measures becomes a random variable, whose precise statistics are of general interest. Indeed, distributions of observables on random graphs constitute an important benchmark, as “null models” constructed out of random interactions can then be compared with empirical data to quantify the effect of structure and “information” encoded in the data that cannot be explained by pure noise.

Perhaps surprisingly, though, the available analytical results for the full distribution of centrality measures on random networks are particularly scarce. This is probably due to the “global” character of most centrality measures, which require complete information about all other nodes to be characterized exactly.

In the recent mathematical literature, most of the existing works concern the distribution of PageRank on directed random graphs (43–51), in particular, aimed at proving rigorously the empirically observed “power-law hypothesis:” In a Scale Free network, the PageRank scores follow a power law with the same exponent as the (in-)degrees (52–56). In this context, the distribution of PageRank was found to obey a distributional fixed-point equation, which seemingly facilitated analytical considerations. However, the derivations are not particularly transparent or illuminating—at least to our eyes—and do not allow easy access to an operational scheme to control and solve the distributional equations. Upper bounds and approximations to the PageRank distribution are provided in ref. 57 for d-regular directed acyclic random networks generated by the configuration model. The distribution of betweenness centrality was considered for exponential random graph models in ref. 58 and for random trees and other subcritical graph families in ref. 59. Exact calculations of centrality vectors for instances of networks with special structures are also available (60). For undirected random graphs, bounds and convergence of the PageRank distribution have been obtained in ref. 61, while numerical explorations of distributions of various centrality measures (including PageRank) as well as analytical results for networks with preferential attachment are presented in ref. 62. For an empirical study of the distribution of centralities in urban settings, see refs. 63 and 64.

In this paper, we focus on the Katz centrality of undirected random networks with N nodes that are locally tree-like, meaning that short loops are rare and the typical size of a loop is O(logN). However, our techniques work also in the case of other similarly constructed centrality measures (65). We aim to characterize analytically the full distribution of the Katz centrality of nodes i) within a single instance with N nodes, and ii) across the entire ensemble of large random graphs with fixed mean degree c for N→∞, focusing on Erdős-Rényi and Scale Free graphs as prominent examples^*^—although the theory works as well for any configuration model characterized by the degree distribution p(k).

Leveraging a fast recursive scheme based on Cavity/Gaussian Belief Propagation (GaBP) to solve linear systems on a tree-like structure (67–69), we first show that the Katz centralities of all nodes of a single instance solve a system of recursive equations for cavity fields, which can be solved very efficiently. Next, we exploit this result to claim that the corresponding distribution of Katz centralities across the entire ensemble can be determined as the solution of a set of recursive distributional equations—essentially, integral equations for probability density functions (pdf). Not only are these equations written out explicitly, but an efficient numerical scheme (Population Dynamics) is proposed to solve them numerically, the only necessary ingredient being the degree distribution p(k) of the network of interest. The numerical solution of the population dynamics scheme is in excellent agreement with numerical simulations of large random networks with fixed average connectivity.

The progress we made in the analytical computation of the probability density of the Katz centrality across nodes having the same degree in the configuration model (see Eq. **38** below) makes it possible to validate the functional importance of nodes in an empirical network against a “null” model where only the information about node degrees is retained. We test this statistical validation scheme on a dataset comprising airline routes connecting 3,425 world airports (70) retrieved from the OpenFlights database, as well as on a Facebook friendship interaction network (71), and a citation network (72) (*SI Appendix*). In all cases, we are able to identify three classes of nodes: those whose centrality is over- or underexpressed relative to the value one might have expected for nodes of the same degree in the “null” model and those whose centrality is instead compatible with their “null” model counterparts.

We also propose an approximate scheme—based on a rank-1 projection of the adjacency matrix proposed in ref. 65 and successfully used in refs. 73 and 74—to reproduce the distribution of Katz centrality for not too sparse graphs, which also works very well. All our results confirm and put on firmer analytical ground the known observations that centrality measures are often correlated with each other (75–78), as we show that the distribution of Katz centrality can be naturally decomposed into contributions coming from nodes of given degree (see Eq. **38** below) yielding a strong correlation between Katz and degree centrality of each node (see Figs. 2 and 3 below).

We will also argue that an extension of our framework is likely to be useful to compute analytically the full distribution of other centrality measures (for example, PageRank in directed graphs) in a transparent and easy-to-interpret way.

The plan of the paper is as follows. In Section 2, we provide the definition and interpretation of Katz centrality, and we show that the centralities of nodes can be computed as the solution of a linear system. In Section 3, we provide a pedagogical derivation of the cavity/BP recursive equations that allow us to solve a sparse linear system of equations on a tree-like structure in a fast and efficient way. In Section 4, we leverage this result to derive a set of recursive equations to compute the Katz centrality of all nodes of a single instance of a network in a fast and distributed way. In Section 5, we exploit these results to show that the full probability distribution P(K) of observing a node with Katz centrality K in an ensemble of large random networks is determined as the solution of a pair of recursive distributional equations, which can be efficiently solved using a Population Dynamics algorithm presented in Section 6 along with the result of numerical simulations. In Section 7, we present the results on empirical validation of the importance of nodes in the airport network dataset against a “null” benchmark where only the information about the degree sequence is retained. In Section 8, we construct an approximate scheme—based on a rank-1 projection of the adjacency matrix—to write P(K) in a more explicit form, which works well in certain conditions. Finally, in Section 9, we offer some concluding remarks and an outlook for future research.

## 2. Katz Centrality

In graph theory, the Katz centrality of a node was first introduced by Leo Katz in 1953 (31) to measure the relative degree of influence of an agent within a social network by taking into account the total number of walks that connect the agent with all the others. Paths connecting an agent with a “distant” node are however penalized by an attenuation factor α.

More formally, let G be the N×N symmetric adjacency matrix of an undirected network formed by N nodes, with $G_{\mathrm{ij}}=G_{\mathrm{ji}}=1$ if node i is connected to node j, and 0 otherwise. The powers of G indicate the presence (or absence) of links between two nodes through intermediaries. For instance, the element ${\left(G^{k}\right)}_{\mathrm{ij}}$ counts the number of paths of length k between nodes i and j.

Given a parameter α∈(0,1), $K_{i}$ denotes the Katz centrality of node i if

[1] $$\begin{array}{c} K_{i}=\sum_{k=1}^{\infty }\sum_{j=1}^{N}\alpha^{k}{\left(G^{k}\right)}_{\mathrm{ji}}\;. \end{array}$$

The interpretation is straightforward: The centrality of a node is a weighted sum of paths of all lengths reaching that node from all other nodes, where longer paths are weighted less—see ref. 79 for proposals on how to optimally select the parameter α.

The value of the attenuation factor α has to be chosen such that

[2] $$\begin{array}{c} 0<\alpha <\frac{1}{\lambda_{\text{max}}}\;, \end{array}$$

where $\lambda_{\text{max}}$ is the largest eigenvalue of G, for the infinite sum in Eq. **1** to converge. Interestingly, it follows from the definition in Eq. **1** that

[3] $$\begin{array}{c} \lim_{\alpha \to 0^{+}}\frac{K_{i}}{\alpha }=k_{i}\;, \end{array}$$

where $k_{i}=\sum_{j}G_{\mathrm{ji}}$ is the degree of node i, i.e. the number of its neighbors. Conversely,

[4] $$\begin{array}{c} \lim_{\alpha \to {\left(1/\lambda_{\text{max}}\right)}^{-}}\left(1-\alpha \lambda_{\text{max}}\right)K_{i}=\xi E_{i}\;, \end{array}$$

where $E_{i}$ is the eigenvector centrality of node i, i.e. the i-th component of the vector E that solves the eigenvector equation $GE=\lambda_{\text{max}}E$, and ξ is a numerical constant; see, e.g., ref. 80.

The infinite geometric sum in Eq. **1** converges to

[5] $$\begin{array}{c} K=\underset{K_{s}}{\underbrace{{\left(1-\alpha G\right)}^{-1}1}}-1\;, \end{array}$$

where 1 is the N×N identity matrix, and 1 is a N-dimensional column vector. Here, K is the vector collecting the N centralities of all nodes. From Eq. **5** and the fact that αG is substochastic, it follows^†^ that $K_{i}\geq 0$.

Rearranging Eq. **5** slightly, we can rewrite the vector of centralities as the solution of the linear system of equations

[6] $$\begin{array}{c} \left(1-\alpha G\right)K_{s}=1\;, \end{array}$$

where $K_{s}=K+1$.

In the following section, we review the algorithm to solve efficiently a linear system of equations on a sparse structure using a recursive method (GaBP/cavity) proposed in refs. 67–69. We initially follow their idea of reframing the calculation of single-instance centrality as the solution of a linear system on a sparse structure, as they first suggested in the case of an extension of Katz centrality to weighted networks that they named spatial ranking (69, 82). Standard iterative schemes for linear systems such as Gauss-Seidel, Jacobi, and conjugate gradient (83) are routinely used to numerically compute the centrality values on a single instance (84), as they are more stable and faster than matrix inversion methods. The GaBP/Cavity scheme has however two main advantages: i) there is some numerical evidence that the GaBP/cavity scheme is superior to standard recursive linear system methods in terms of performances and stability on sparse structures (85, 86), and ii) contrary to classical recursive methods, the GaBP/Cavity scheme provides explicit equations connecting single-instance node and edge fields, which can be easily translated into analytical distributional equations at the ensemble level. We start in the next section by presenting the general GaBP/cavity theory for the solution of sparse linear systems.

## 3. Solution of a Sparse Linear System with the Cavity Method

Consider a linear system

[7] $$\begin{array}{c} Ax=b, \end{array}$$

with A square, symmetric and invertible. The fundamental observation is that the solution vector

[8] $$\begin{array}{c} x^{⋆}=A^{-1}b, \end{array}$$

is identical to the vector of averages

[9] $$\begin{array}{c} x_{i}^{*}=\mu_{i}=\int dx_{i}\;x_{i}p_{i}\left(x_{i}\right)\;, \end{array}$$

with

[10] $$\begin{array}{c} p_{i}\left(x_{i}\right)=\int \prod_{j\neq i}dx_{j}\;p\left(x\right), \end{array}$$

and p(x) given by the following multivariate Gaussian

[11] $$\begin{array}{c} p\left(x\right)=\frac{1}{Z}\exp\left[-\frac{1}{2}x^{T}Ax+b^{T}x\right]\;. \end{array}$$

This follows from

[12] $$\begin{array}{c} {\left(x-x^{⋆}\right)}^{T}A\left(x-x^{⋆}\right)=x^{T}Ax-2b^{T}x+b^{T}A^{-1}b\;, \end{array}$$

which allows us to write the multivariate Gaussian with mean vector $x^{⋆}$ in the form of Eq. **11**

[13] $$\begin{array}{cc} p(x) & =\frac{1}{Z^{\prime }}\exp\left[-\frac{1}{2}{\left(x-x^{⋆}\right)}^{T}A\left(x-x^{⋆}\right)\right]\\ & =\frac{1}{Z}\exp\left[-\frac{1}{2}x^{T}Ax+b^{T}x\right]\;, \end{array}$$

with $Z=Z^{\prime }\exp\left[\left(1/2\right)b^{T}A^{-1}b\right]$.

Writing the solution in the form of Eq. **9** transfers the problem from the linear algebra domain to the probabilistic domain, allowing us to tackle it with a more powerful and broader set of tools.

From now on, we further assume that the matrix A of coefficients of the linear system defines a locally tree-like graph structure, where the unknowns $x_{i}$ live on the N nodes of a graph, and the coefficients $A_{\mathrm{ij}}\neq 0$ stand for the weight of the edge connecting node i and j.

If the graph is a tree—but the treatment below works very well for tree-like structures—we can appeal to the GaBP scheme (67–69)—a particular incarnation of the cavity method (87–89) from the theory of disordered systems, and of message passing algorithms (90–92)—to find efficient and fast recursive equations for the averages $\mu_{i}$ we are after. Among the many virtues of the scheme is the fact that—when the algorithm converges—it is guaranteed to converge to the true averages (i.e. the inference is guaranteed to be exact) (67, 69). In our case, the convergence of the algorithm follows from the condition of Eq. **2**, which defines a walk-summable problem (see ref. 93, Proposition 2).

Let us start by rewriting the marginal $p_{i}\left(x_{i}\right)$ as follows

[14] $$\begin{array}{cc} p_{i}\left(x_{i}\right) & =\frac{1}{Z_{i}}\int \prod_{j\neq i}dx_{j}\exp\left[-\frac{1}{2}\sum_{i}x_{i}\sum_{j\in \partial i}A_{\mathrm{ij}}x_{j}+\sum_{k}b_{k}x_{k}\right]\\ & =\frac{1}{Z_{i}}e^{-\frac{1}{2}A_{\mathrm{ii}}x_{i}^{2}+b_{i}x_{i}}\int \prod_{j\in \partial i}dx_{j}\exp\left[-x_{i}\sum_{j\in \partial i}A_{\mathrm{ij}}x_{j}\right]\\ & \;\times \;p^{\left(i\right)}\left(x_{\partial i}\right)\;, \end{array}$$

where ∂i denotes the set of nodes j connected to i ($A_{\mathrm{ij}}\neq 0$), while $p^{\left(i\right)}\left(x_{\partial_{i}}\right)$ denotes the cavity distribution, namely the joint distribution of remaining variables (so, from the j-th variable outward) after the node i has been removed from the picture.

**Fig. 1.**
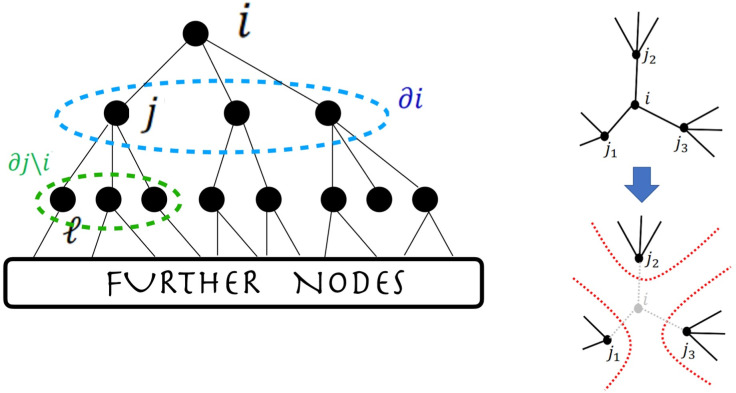
Sketch of the tree structure with the node i on top, the neighborhood ∂i in dashed blue, and the further-down neighborhood ∂j∖i in dashed green (*Left*). On the *Right*, schematic representation of the removal of node i, which leaves nodes $j_{1}$, $j_{2}$, and $j_{3}$ independent.

Now, in a tree structure, the nodes j in the neighborhood of i are only connected to each other via the node i (see sketch in Fig. 1). When the node i is removed, the variables defined on these nodes become therefore independent, i.e. the cavity distribution factorizes over the nodes in the neighborhood of i

[15] $$\begin{array}{c} p^{\left(i\right)}\left(x_{\partial i}\right)=\prod_{j\in \partial i}p_{j}^{\left(i\right)}\left(x_{j}\right)\;. \end{array}$$

Therefore

[16] $$\begin{array}{cc} p_{i}\left(x_{i}\right) & =\frac{1}{Z_{i}}e^{-\frac{1}{2}A_{\mathrm{ii}}x_{i}^{2}+b_{i}x_{i}}\\ & \;\times \prod_{j\in \partial i}\int dx_{j}\exp\left[-x_{i}A_{\mathrm{ij}}x_{j}\right]p_{j}^{\left(i\right)}\left(x_{j}\right)\;. \end{array}$$

We can repeat the reasoning for the cavity distribution itself

[17] $$\begin{array}{cc} p_{j}^{\left(i\right)}\left(x_{j}\right) & =\frac{1}{Z_{j}^{\left(i\right)}}e^{-\frac{1}{2}A_{\mathrm{jj}}x_{j}^{2}+b_{j}x_{j}}\\ & \;\times \prod_{\ell \in \partial j\setminus i}\int dx_{\ell }\exp\left[-x_{j}A_{j\ell }x_{\ell }\right]p_{\ell }^{\left(j\right)}\left(x_{\ell }\right)\;, \end{array}$$

where ∂j∖i denotes the set of neighbors of node j excluding the node i. Note that Eq. **17** is now a closed recursion for the cavity distributions $p_{j}^{\left(i\right)}$, whereas Eq. **16** is not a closed recursion for the marginal $p_{i}\left(x_{i}\right)$. Knowing the cavity marginals (solutions of Eq. **17**), though, it is possible to compute the marginals using Eq. **16**, as we show below.

We make the (normalized) Gaussian ansatz for the cavity distribution

[18] $$\begin{array}{c} p_{j}^{\left(i\right)}\left(x\right)=\frac{1}{Z_{j}^{\left(i\right)}}\exp\left(-\frac{{\left(x-\mu_{j}^{\left(i\right)}\right)}^{2}}{2V_{j}^{\left(i\right)}}\right), \end{array}$$

with cavity mean $\mu_{j}^{\left(i\right)}$ and cavity variance $V_{j}^{\left(i\right)}$. With this choice, the integral on the r.h.s. of Eq. **17** is Gaussian, which in turn will result in a Gaussian dependence on $x_{j}$. Inserting this ansatz on the r.h.s. of Eq. **17**, we compute the resulting Gaussian integral using the formula

[19] $$\begin{array}{c} \left\langle e^{-Mx}\right\rangle =e^{\frac{M^{2}V}{2}-M\mu }\;, \end{array}$$

where ⟨·⟩ stands for averaging over a normalized Gaussian N(μ,V) with mean μ and variance V. Specializing to

[20] $$\begin{array}{c} M=x_{j}A_{j\ell }, \end{array}$$

from Eq. **17**, we see that the exponent in the r.h.s. becomes

[21] $$\begin{array}{c} -\frac{1}{2}x_{j}^{2}\left(A_{\mathrm{jj}}-\sum_{\ell \in \partial j\setminus i}V_{\ell }^{\left(j\right)}A_{j\ell }^{2}\right)+x_{j}\left(b_{j}-\sum_{\ell \in \partial j\setminus i}A_{j\ell }\mu_{\ell }^{\left(j\right)}\right)\;. \end{array}$$

Furthermore, the average and variance of a normalized Gaussian of the form appearing in the r.h.s. of Eq. **17**, namely

[22] $$\begin{array}{c} p\left(x\right)=\frac{1}{Z}e^{-\frac{1}{2}Cx^{2}+Dx}, \end{array}$$

are respectively

[23] $$\begin{array}{cc} V & =\frac{1}{C}\;, \end{array}$$

[24] $$\begin{array}{cc} \mu & =\frac{D}{C}=DV\;. \end{array}$$

Using the expressions above, we get—equating mean and variance—from Eq. **17** and using the ansatz Eq. **18**

[25] $$\begin{array}{cc} V_{j}^{\left(i\right)} & =\frac{1}{A_{\mathrm{jj}}-\sum_{\ell \in \partial j\setminus i}V_{\ell }^{\left(j\right)}A_{j\ell }^{2}}, \end{array}$$

[26] $$\begin{array}{cc} \mu_{j}^{\left(i\right)} & =V_{j}^{\left(i\right)}\left(b_{j}-\sum_{\ell \in \partial j\setminus i}A_{j\ell }\mu_{\ell }^{\left(j\right)}\right)\;. \end{array}$$

Similarly, we make the (normalized) Gaussian ansatz for the marginal distribution

[27] $$\begin{array}{c} p_{j}\left(x\right)=\frac{1}{Z_{j}}\exp\left(-\frac{{\left(x-\mu_{j}\right)}^{2}}{2V_{j}}\right), \end{array}$$

with mean $\mu_{j}$ and variance $V_{j}$. Inserting again the Gaussian ansatz of Eq. **18** for the cavity marginal in the r.h.s. of Eq. **16**, and comparing with the ansatz Eq. **27** for the l.h.s., we obtain the following equations

[28] $$\begin{array}{cc} V_{j} & =\frac{1}{A_{\mathrm{jj}}-\sum_{\ell \in \partial j}V_{\ell }^{\left(j\right)}A_{j\ell }^{2}}\;, \end{array}$$

[29] $$\begin{array}{cc} \mu_{j} & =V_{j}\left(b_{j}-\sum_{\ell \in \partial j}A_{j\ell }\mu_{\ell }^{\left(j\right)}\right)\;. \end{array}$$

Solving the self-consistency Eqs. **25** and **26** on the cavity graph and inserting the results into Eqs. **28** and **29** provides the solution $x_{i}^{⋆}=\mu_{i}$ of the linear system Eq. **7**. The equations above are identical to those provided in ref. 68, after some rewriting and rearrangements. In the next section, we are going to specialize these results to the case of the linear system Eq. **6** defining the shifted Katz centrality on a single network instance.

## 4. Katz Centrality on Single Instance of a Random Graph

To apply the formalism developed in the previous section to the Katz centrality, we may define from Eq. **6** the matrix A as

[30] $$\begin{array}{c} A_{j\ell }=\delta_{j\ell }-\alpha G_{j\ell }=\left\{\begin{array}{cc} -\alpha & \;\;\text{if}j\neq \ell \\ 1 & \;\;\text{if}j=\ell \end{array}\right.\;, \end{array}$$

since we assume that a link exists between node j and ℓ, and that there are no self-loops. Also, $b_{j}=1$ for all j.

The self-consistent cavity equations thus become

[31] $$\begin{array}{cc} V_{j}^{\left(i\right)} & =\frac{1}{1-\alpha^{2}\sum_{\ell \in \partial j\setminus i}V_{\ell }^{\left(j\right)}}\;, \end{array}$$

[32] $$\begin{array}{cc} \mu_{j}^{\left(i\right)} & =V_{j}^{\left(i\right)}\left(1+\alpha \sum_{\ell \in \partial j\setminus i}\mu_{\ell }^{\left(j\right)}\right), \end{array}$$

[33] $$\begin{array}{cc} V_{j} & =\frac{1}{1-\alpha^{2}\sum_{\ell \in \partial j}V_{\ell }^{\left(j\right)}}\;, \end{array}$$

[34] $$\begin{array}{cc} \mu_{j} & =V_{j}\left(1+\alpha \sum_{\ell \in \partial j}\mu_{\ell }^{\left(j\right)}\right)\;, \end{array}$$

from which the Katz centrality $K_{i}$ of node i can be efficiently determined from Eq. **6** as

[35] $$\begin{array}{c} K_{i}=\mu_{i}-1\;. \end{array}$$

**Fig. 2.**
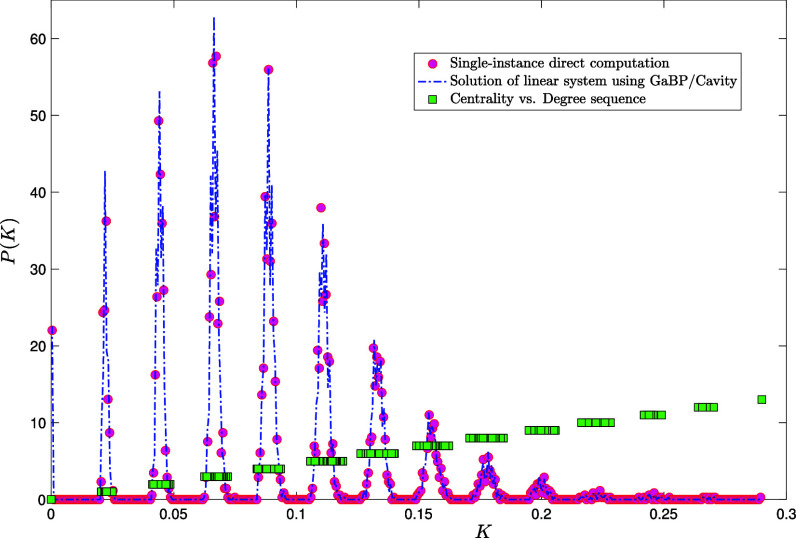
Probability density function P(K) of the Katz centrality with α=1/50 computed over a single instance of an Erdős-Rényi graph of size N=5,000 with average degree c=4 by direct matrix inversion from Eq. **5** (pink circles). Blue dot-dashed line: GaBP/cavity solution of the linear system as given in Eqs. **31**–**35**. The coordinates $\left(K_{j},k_{j}\right)$ of each green square j=1,…,N provide the degree $k_{j}$ of node j against its centrality $K_{j}$.

**Fig. 3.**
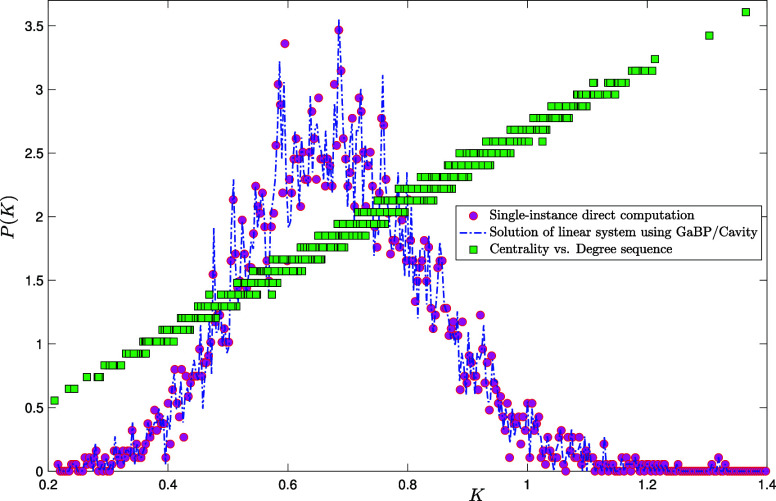
Probability density function P(K) of the Katz centrality with α=1/50 computed over a single instance of an Erdős-Rényi graph of size N=5,000 with average degree c=20 by direct matrix inversion from Eq. **5** (pink circles). Blue dot-dashed line: GaBP/cavity solution of the linear system as given in Eqs. **31**–**35**. The coordinates $\left(K_{j},k_{j}/m\right)$ of each green square j=1,…,N provide the degree $k_{j}$ of node j—rescaled by a factor m=10.81 to make it visible on the same scale—against its centrality $K_{j}$.

In Fig. 2, we plot the Katz centrality distribution for a single instance of an Erdős-Rényi graph of size N=5,000 with average degree c=4, along with the GaBP/Cavity solution of the recursions above, as well as the degree sequence staircase (green squares). From the plot, one easily infers that the centrality distribution is naturally decomposed into contributions (peaks) coming from nodes of different degrees. Increasing the average connectivity c, the peaks would gradually merge, as more and more nodes of different degrees happen to have the same centrality (see Fig. 3 for c=20).

## 5. Probability *P*(*K*) Over the Ensemble

We are now interested in leveraging the results of the previous section—valid for a single instance of a random network—to compute the probability density function P(K) of finding a node i with centrality $P\left(K\right)=\mathrm{Prob}\left[K_{i}=K\right]$ in an ensemble of large undirected random graphs. Going from single-instance cavity results to distributions over an ensemble is a quite standard procedure (see ref. 94 for a review), which we report here for completeness.

First, one has to focus on the joint probability density function π(μ,V) of observing a cavity mean $\mu_{j}^{\left(i\right)}=\mu$ and a cavity variance $V_{j}^{\left(i\right)}=V$ in the ensemble. To do so, one observes that the self-consistency equations for the cavity variance and mean (Eqs. **31** and **32**) refer to the links of the underlying graph. In an infinitely large network, links can be distinguished from one another by the degree of the node they are pointing to. Therefore, considering an edge (i,j) pointing to a node j of degree k, the value (μ,V) of the pair formed by the cavity mean $\mu_{j}^{\left(i\right)}$ and the cavity variance $V_{j}^{\left(i\right)}$—both living on this edge—is determined by the set ${\left\{\mu_{\ell },V_{\ell }\right\}}_{k-1}$ of the k−1 values of the pair $\left(\mu_{\ell }^{\left(j\right)},V_{\ell }^{\left(j\right)}\right)$ living on each of the edges connecting j with its neighbors ℓ∈∂j\i. In an infinite system, these values can be regarded as k−1 independent realizations of the pair of random variables of type $\mu_{\ell }^{\left(j\right)}$ and $V_{\ell }^{\left(j\right)}$, each drawn from the same joint pdf π(μ,V).

The joint pdf π(μ,V) is then obtained by averaging the contributions coming from every link w.r.t. the probability $\frac{k}{c}p\left(k\right)$ of having a link pointing to a node of degree k,^‡^ with p(k) being the degree distribution of the network, and c∼O(1) the average connectivity. This reasoning leads to the self-consistency equation

[36] $$\begin{array}{cc} \pi (\mu ,V) & =\sum_{k=1}^{\infty }p\left(k\right)\frac{k}{c}\int {\left\{d\pi \right\}}_{k-1}\delta \left(\mu -V\left(1+\alpha \sum_{\ell =1}^{k-1}\mu_{\ell }\right)\right)\\ & \;\times \delta \left(V-\frac{1}{1-\alpha^{2}\sum_{\ell =1}^{k-1}V_{\ell }}\right)\;, \end{array}$$

where ${\left\{d\pi \right\}}_{k-1}=\prod_{\ell =1}^{k-1}d\mu_{\ell }dV_{\ell }\pi \left(\mu_{\ell },V_{\ell }\right)$. The recursive distributional Eq. **36** can be efficiently solved via a population dynamics algorithm (Section 6). Note that the integral equations above can now be considered and solved independently of the network problem that originated them, since no other information about the topology of such network enters the picture apart from the degree distribution p(k), which makes this approach so general and powerful.

The same reasoning can be applied to find the joint pdf $\tilde{\pi }\left(\tilde{\mu },\tilde{V}\right)$ of the pair $\left(\mu_{i},V_{i}\right)$ satisfying Eqs. **33** and **34**. From there, one notices that the $\mu_{i}$ and $V_{i}$ are variables related to nodes, rather than edges. Since in the infinite size limit the nodes can be distinguished from one another by their degree, the joint pdf $\tilde{\pi }\left(\tilde{\mu },\tilde{V}\right)$ can be written in terms of the solution π(μ,V) of Eq. **36** as

[37] $$\begin{array}{cc} \tilde{\pi }\left(\tilde{\mu },\tilde{V}\right) & =\sum_{k=0}^{\infty }p\left(k\right)\int {\left\{d\pi \right\}}_{k}\delta \left(\tilde{\mu }-\tilde{V}\left(1+\alpha \sum_{\ell =1}^{k}\mu_{\ell }\right)\right)\\ & \;\times \delta \left(\tilde{V}-\frac{1}{1-\alpha^{2}\sum_{\ell =1}^{k}V_{\ell }}\right)\;, \end{array}$$

where p(k) is again the degree distribution. Note that the r.h.s. of Eq. **37** is a sum of k-fold integrals involving π and not $\tilde{\pi }$, because $\mu_{i}$ and $V_{i}$ are defined in terms of the “cavity” pair (Eqs. **33** and **34**). Also, the integral relations above evidently preserve the normalization of the joint pdfs π and $\tilde{\pi }$.

After solving Eq. **37** for the joint pdf $\tilde{\pi }\left(\tilde{\mu },\tilde{V}\right)$ of the variables of type $\mu_{i}$ and $V_{i}$, we appeal to Eq. **35** and the definition of the shifted Katz centrality as a linear system in Eq. **6** to write the pdf $P(K_{s})$ as

[38] $$\begin{array}{c} P\left(K_{s}\right)=\int d\tilde{V}\;\tilde{\pi }\left(K_{s},\tilde{V}\right)\;=\sum_{k=0}^{\infty }p\left(k\right)P\left(K_{s}|k\right)\;, \end{array}$$

with the pdf $P(K_{s}|k)$ of a node having shifted centrality $K_{s}$ given that it has degree k given by

[39] $$\begin{array}{cc} & P(K_{s}|k)\\ & \;\;=\int {\left\{d\pi \right\}}_{k}\delta \left(K_{s}-\left(\frac{1}{1-\alpha^{2}\sum_{\ell =1}^{k}V_{\ell }}\right)\left(1+\alpha \sum_{\ell =1}^{k}\mu_{\ell }\right)\right). \end{array}$$

**Fig. 4.**
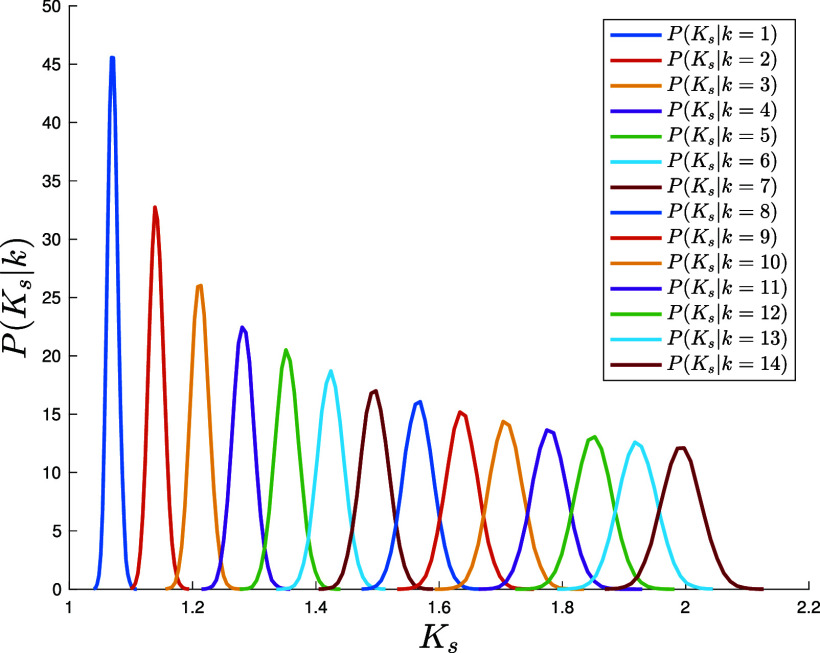
Conditional pdf $P(K_{s}|k)$ of the shifted Katz centrality $K_{s}=K+1$ of nodes of degree k for an ensemble of graphs with Poissonian degree distribution with mean c=35, α=1/40 and population size $N_{P}=10^{5}$ (Eq. **38**). The curves are obtained via Monte Carlo sampling of the integral in Eq. **39** after the population has reached equilibrium. We display curves for degree up to k=14.

**Fig. 5.**
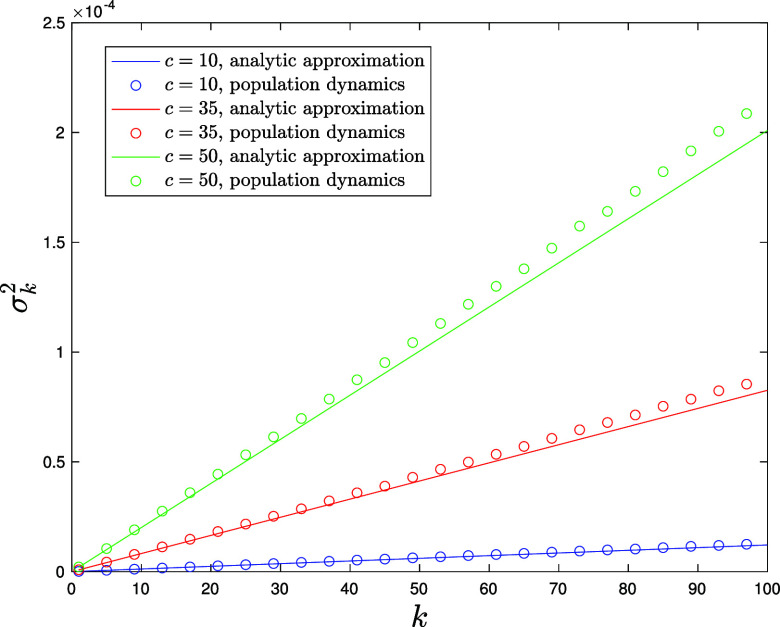
Variance $\sigma_{k}^{2}$ of the Katz centrality distribution (α=0.01) conditioned on nodes of degree k for ensembles of graphs with Poissonian degree distribution with mean c=10 (blue circles), c=35 (red circles), and c=50 (green circles). The variance is computed averaging $K_{s}^{2}$ over the conditional distribution in Eq. **39**, which is in turn computed via Monte Carlo sampling from the equilibrium distribution π [solution of Eq. **36** with Poissonian p(k)]. The corresponding solid lines represent an approximate but explicit formula for this linear trend, which we derive in *SI Appendix* after postulating a suitable simple ansatz for the solution π of Eq. **36**, and a continuum approximation for the degree k. The approximate formula captures the numerical trend reasonably well, even for values of k well beyond the expected range of validity, with noticeable discrepancies only for very large k compared to the mean degree c.

The integral in Eq. **39** can be estimated via Monte Carlo sampling from the equilibrium distribution π(μ,V) in Eq. **36**. Written as in Eq. **38**, the pdf of the Katz centrality is naturally expressed as a superposition of contributions, each coming from nodes of degree k (see Fig. 4 for an ensemble of graphs with Poissonian degree distribution with mean c=35). For sufficiently low average connectivity c, the individual degree-k contributions are clearly visible in the form of distinct peaks. Empirical conditional probability distributions for centrality measures such as the betweenness have been previously analyzed to characterize correlations of such metrics with the degree as well as their expected behavior compared to random models (95). In Fig. 5, we plot the variance $\sigma_{k}^{2}$ of the conditional distribution $P(K_{s}|k)$ of the shifted Katz centrality as a function of the node’s degree for a Poissonian ensemble of graphs with mean degree c=10 (blue), c=35 (red), and c=50 (green) together with an analytic approximate formula (solid lines) valid in principle for large c, small α, and $k=c\pm O(\sqrt{c})$ (*SI Appendix*). For the range of parameters considered in the figure, the analytic approximation—obtained postulating a suitable ansatz for the function π(μ,V) in Eq. **36** and a continuum approximation for the degree k—captures the essential trend of the variance quite accurately and even beyond the a priori expected range of validity for k. We observe that the variance is increasing linearly with the node’s degree, with a steeper slope for larger average connectivity c, a feature that is perfectly captured by the approximate analytic formula (*SI Appendix*).

## 6. Numerical Solution Using Population Dynamics

In this section, we describe the stochastic population dynamics algorithm that leads to the solution of the self-consistency Eq. **36** for the joint pdf π(μ,V), coupled with the sampling procedure to evaluate Eq. **37**. This kind of algorithm is widely used in the study of amorphous systems (96), spin glasses (97, 98), random matrices (94, 99–101) and percolation in sparse networks (102).

First, in order to solve Eq. **36**, one represents the joint pdf π(μ,V) in terms of two populations of $N_{P}$ ordered real values, $M\equiv \{\mu_{1},\ldots ,\mu_{N_{P}}\}$ and $V\equiv \{V_{1},\ldots ,V_{N_{P}}\}\geq 0$, which are assumed to be sampled from that joint pdf. Given that the true jpdf is initially unknown, a starting population of pairs (M,V) is initialized randomly.

Similarly, one represents the joint pdf $\tilde{\pi }\left(\tilde{\mu },\tilde{V}\right)$ in terms of two populations of $N_{P}$ ordered real values, $\tilde{M}\equiv \left\{{\tilde{\mu }}_{1},\ldots ,{\tilde{\mu }}_{N_{P}}\right\}$ and $\tilde{V}\equiv \left\{{\tilde{V}}_{1},\ldots ,{\tilde{V}}_{N_{P}}\right\}\geq 0$, which are assumed to be sampled from that joint pdf. Again, a starting population of pairs $\left(\tilde{M},\tilde{V}\right)$ is initialized randomly.

Then the following stochastic algorithm is iterated until two stable populations are reached. 1) Generate a random integer k from the distribution $\frac{k}{c}p\left(k\right)$, where p(k) is the degree distribution of the ensemble of interest and $c=\sum_{k}kp\left(k\right)$ is the average degree; 2) Generate a random integer $\tilde{k}$ from the degree distribution p(k); 3) Select k−1 pairs of values $\left(\mu_{\ell }^{\text{(old)}},V_{\ell }^{\text{(old)}}\right)$ at random—sharing the same indices—from the two populations (M,V) respectively; 4) Select $\tilde{k}$ pairs of values $\left({\tilde{\mu }}_{\ell }^{\text{(old)}},{\tilde{V}}_{\ell }^{\text{(old)}}\right)$ at random—sharing the same indices—from the two populations $\left(\tilde{M},\tilde{V}\right)$ respectively; 5) Compute the new values^§^

[40] $$\begin{array}{cc} V^{\text{(new)}} & =\frac{1}{1-\alpha^{2}\sum_{\ell =1}^{k-1}V_{\ell }^{\text{(old)}}}\;, \end{array}$$

[41] $$\begin{array}{cc} \mu^{\text{(new)}} & =V^{\text{(new)}}\left(1+\alpha \sum_{\ell =1}^{k-1}\mu_{\ell }^{\text{(old)}}\right), \end{array}$$

[42] $$\begin{array}{cc} {\tilde{V}}^{\text{(new)}} & =\frac{1}{1-\alpha^{2}\sum_{\ell =1}^{\tilde{k}}V_{\ell }^{\text{(old)}}}\;, \end{array}$$

[43] $$\begin{array}{cc} {\tilde{\mu }}^{\text{(new)}} & ={\tilde{V}}^{\text{(new)}}\left(1+\alpha \sum_{\ell =1}^{\tilde{k}}\mu_{\ell }^{\text{(old)}}\right)\;. \end{array}$$

6) Replace a randomly selected element $V^{\text{(old)}}$ of V with $V^{\text{(new)}}$, and the element $\mu^{\text{(old)}}$ of M with the same index as $V^{\text{(old)}}$ with $\mu^{\text{(new)}}$. 7) Replace a randomly selected element ${\tilde{V}}^{\text{(old)}}$ of $\tilde{V}$ with ${\tilde{V}}^{\text{(new)}}$, and the element ${\tilde{\mu }}^{\text{(old)}}$ of $\tilde{M}$ with the same index as ${\tilde{V}}^{\text{(old)}}$ with ${\tilde{\mu }}^{\text{(new)}}$. 8) Return to 1.

Once two stable populations are reached, the pdf of the shifted centrality is simply obtained by histogramming the population $\tilde{M}$. The fact that the populations have reached convergence is established by monitoring the first and second moments of the samples and stopping when they have clearly plateaued.

In the following, we show the comparison between the numerical solution obtained with population dynamics and direct matrix inversion for Erdős-Rényi and Scale Free networks. Erdős-Rényi networks were built by drawing each possible link with the same probability p=c/(N−1), which leads to networks with a Poisson degree distribution in the limit of large N. Scale Free networks were built using the uncorrelated configuration model (104): Each node was assigned a number of half-links drawn from a power law distribution $P\left(k\right)\propto k^{-\gamma }$, and these were randomly matched to form links. We also check that the occurrence of multiple links and self-loops is avoided. Furthermore, to prevent degree correlations we imposed a cut-off to the degree sequence so that the maximum allowed degree is $\sqrt{k_{\;\text{min}}N}$, with $k_{\;\text{min}}$ being the minimum degree.

**Fig. 6.**
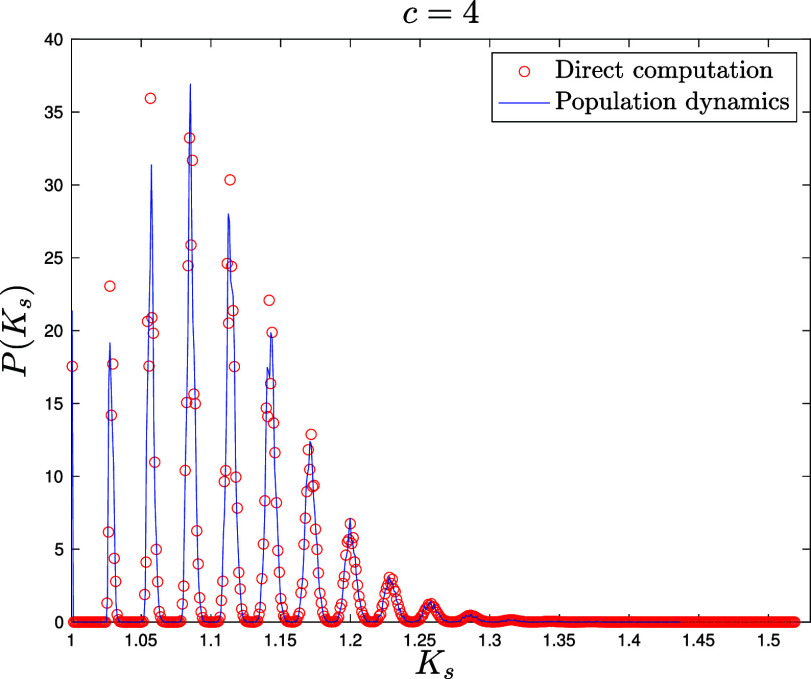
Probability density function $P(K_{s})$ of the shifted Katz centrality with α=1/40 computed over an ensemble of 1,000 Erdős-Rényi graphs of size N=1,000 with average degree c=4 by direct matrix inversion from Eq. **5** (red circles). Blue solid line: distribution of the population $\tilde{M}$ after reaching equilibrium, with $N_{P}=10^{5}$ population members and 100 updating sweeps (see Section 6 for details).

**Fig. 7.**
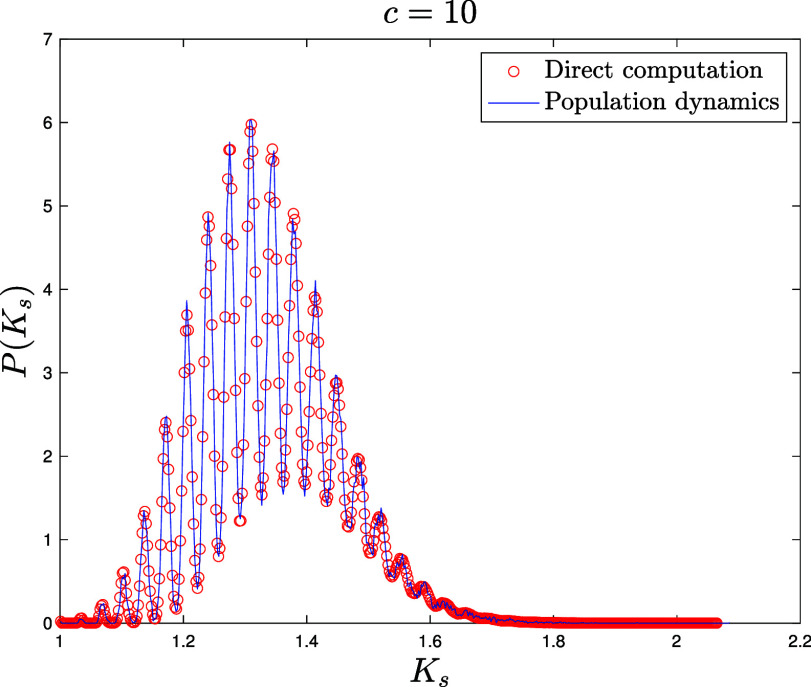
Probability density function $P(K_{s})$ of the shifted Katz centrality with α=1/40 computed over an ensemble of 1,000 Erdős-Rényi graphs of size N=1,000 with average degree c=10 by direct matrix inversion from Eq. **5** (red circles). Blue solid line: distribution of the population $\tilde{M}$ after reaching equilibrium, with $N_{P}=10^{5}$ population members and 100 updating sweeps (see Section 6 for details).

**Fig. 8.**
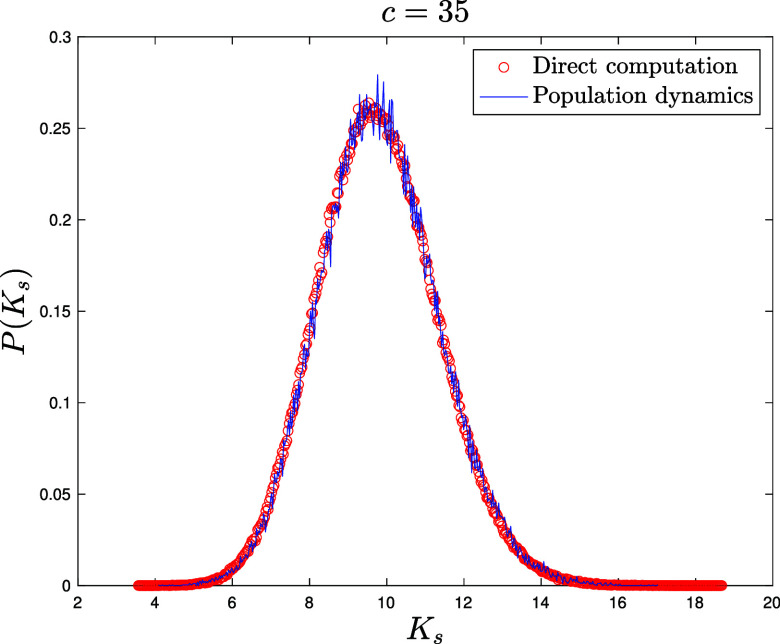
Probability density function $P(K_{s})$ of the shifted Katz centrality with α=1/40 computed over an ensemble of 100 Erdős-Rényi graphs of size N=1,0000 with average degree c=35 by direct matrix inversion from Eq. **5** (red circles). Blue solid line: distribution of the population $\tilde{M}$ after reaching equilibrium, with $N_{P}=10^{5}$ population members and 100 updating sweeps (see Section 6 for details).

**Fig. 9.**
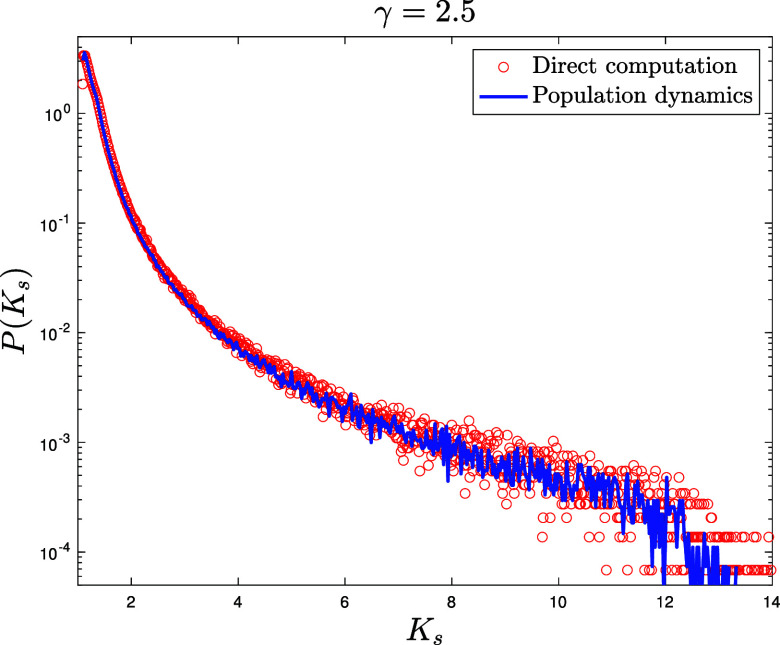
Probability density function $P(K_{s})$ in semilogarithmic scale of the shifted Katz centrality with α=1/40 computed over an ensemble of 100 Scale Free graphs of size N=10,000 with parameter γ=2.5 and minimum degree $k_{\;\text{min}}=3$ by direct matrix inversion from Eq. **5** (red circles). Blue solid line: distribution of the population $\tilde{M}$ after reaching equilibrium, with $N_{P}=10^{6}$ population members and 100 updating sweeps (see Section 6 for details).

To produce the figures below, we use the following parameters:

- for E-R networks (Figs. 6–8) $N_{P}=10^{5}$ for the population dynamics, and 100 sweeps (meaning that each population member has been updated 100 times on average), with α=1/40 and different values c=4,10,35 for the average connectivity. We also perform direct matrix inversion on the adjacency matrices of 1,000 E-R networks of size N=1,000 for c=4,10, while for c=35 we averaged over 100 networks of size N=10,000.
- for Scale Free networks (Fig. 9 and two other figures included in *SI Appendix*) $N_{P}=10^{6}$ for the population dynamics, and 100 sweeps, with α=1/40. The network parameters are γ=2.5 for Fig. 9 and γ=3,4 for the figures in *SI Appendix*, with minimal degree $k_{\;\text{min}}=3$ and degree cutoff at $\sqrt{Nk_{\;\text{min}}}$ to ensure no correlation between degrees (104). We perform direct matrix inversion on the adjacency matrices of 100 Scale Free networks of size N=10,000.

## 7. Statistical Validation on Empirical Networks

In this section, we show how the conditional distribution $P(K_{s}|k)$ derived in Eq. **39** can be exploited in the analysis of empirical networks. As an example, we consider the Global Air Transportation Network retrieved from OpenFlights (see details in *SI Appendix*) comprising 3,425 airports across the globe (70). For each airport having k airline routes departing or arriving, we are interested in determining whether its Katz centrality is significantly higher or lower than the value predicted for nodes of degree k by a suitably constructed random “null” model. This approach will prove very useful in providing information on the functional role played by each airport at different scales (determined by the number of airline routes passing through each node).

The algorithm reads as follows. i) From the degree sequence of the network, we construct the empirical degree distribution $p_{\mathrm{emp}}\left(k\right)=N_{k}/N$, where $N_{k}$ is the number of nodes of degree k that appear in the network. ii) We use $p_{\mathrm{emp}}\left(k\right)$ as p(k) to solve Eqs. **36** and **37** using the population dynamics algorithm described in Section 6. This is equivalent to considering an ensemble of networks where links are randomly rewired, while preserving the degree of each node of the empirical network. iii) For each degree k, we estimate the conditional distribution $P(K_{s}|k)$ in Eq. **39** for nodes of degree k in the null model. iv) From this distribution, we compute the confidence bounds at a given confidence level β and introduce a Bonferroni correction to account for multiple hypothesis testing ($\tilde{\beta }=\beta /N_{k}$). v) For a given confidence level $\tilde{\beta }$ and for a class of nodes of degree k, we identify over- and underexpressed nodes, whose centrality significantly deviates from the null benchmark.

**Fig. 10.**
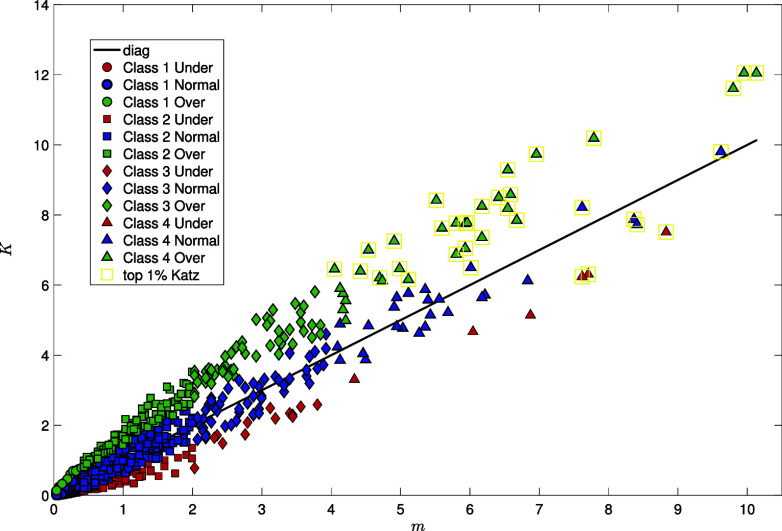
Katz centrality (computed with α=1/90) vs. average centrality conditional on degree for nodes in the Global Transportation Network (N=3,425) (70). Symbols represent different degree classes: Class 1 = degree [1 to 10], Class 2 = degree [11 to 50], Class 3 = degree [51 to 100], and Class 4 = degree 100+. Colors reflect under- (red), normal (blue), and over- (green) expressed categories in terms of Katz centrality for a confidence level β=0.99. Yellow boxes are used to highlight airports whose Katz centrality falls within the top 1%.

The results for the air transportation network are presented in Fig. 10 (validation on other empirical networks are presented in *SI Appendix*). We group the airports in four degree classes, from those with a number of departing/arriving flights between 1 and 10 to those with a number of 100+ direct connections. Our results show that airports with similar degrees can nonetheless differ significantly in their network importance and functionality (see full classification table in *SI Appendix*). For instance, in the category of “small” airports (degree [1 to 10]), underexpressed nodes are mostly regional airports located in isolated or less accessible regions (e.g., remote parts of Alaska, Canada, and Greenland), with mainly domestic coverage and low passengers’ volume. Overexpressed nodes correspond instead to airports in strategic locations, despite having low degree centrality. In particular, they are located in popular (remote) holiday spots, hence acting as significant hubs for their region despite fewer direct connections (e.g., BDA—Bermuda). The remaining nodes offer balanced connectivity, as expected for their size and region, without being overly crucial or underused. Similarly, in the 100+ degree centrality category, airports with underexpressed Katz centrality, such as DME (Moscow Domodedovo, Russia) and ATL (Atlanta, USA), manage high passenger volumes and provide robust regional connectivity. However, their global integration is not so pronounced. On the other hand, airports with overexpressed Katz centrality, such as AMS (Amsterdam, Netherlands) and JFK (New York, USA), are pivotal in the global network. These airports have influential connections that make them essential nodes for supporting global connectivity and regional economic development. Simply ranking nodes by Katz centrality value and selecting a fraction of the top ones as leading hubs of the network, does not provide any information on the finer scale structure. Indeed, the top 1% nodes by Katz centrality coincide with high-degree nodes (class 4, k=[101,248]) and include both under-, over-, and normal nodes as per our importance validation labeling.

Therefore, the validation procedure proposed here can provide more detailed and nuanced information about the functional role played by nodes at different scales, which is not simply captured by their degree.

## 8. Centrality Distribution from Rank-1 Approximation

In this section, we consider the rank-1 approximation to centrality measures proposed in ref. 65, and we show that it leads to an approximate but explicit formula for the distribution $P(K_{s})$, which works very well for c sufficiently high.

The idea is to replace the symmetric adjacency matrix G featuring in Eq. **5** with a rank-1 approximation $\hat{G}$ defined as

[44] $$\begin{array}{c} \hat{G}=\frac{1}{\overset{¯}{k}N}kk^{T}\;, \end{array}$$

where $k={\left\{k_{1},\ldots ,k_{N}\right\}}^{T}$ is the degree sequence of the network represented by G, arranged in a column vector, and $\overset{¯}{k}$ is the mean degree $\frac{1}{N}\sum_{i}k_{i}$. Constructed in this way, the matrix $\hat{G}$ is rank-1 and has the same degree sequence (row sums) of the original matrix G. From Eq. **5**, replacing G with $\hat{G}$ and using the Sherman–Morrison formula (105) to compute the inverse matrix, we obtain

[45] $$\begin{array}{cc} K_{s}≃{\left(1-\alpha \hat{G}\right)}^{-1}1 & =\left(1+\frac{\alpha \hat{G}}{1-\alpha \frac{\sum_{i}k_{i}^{2}}{\sum_{i}k_{i}}}\right)1\\ & =1+\frac{\alpha }{1-\alpha \frac{\sum_{i}k_{i}^{2}}{\sum_{i}k_{i}}}k\;. \end{array}$$

Note that this rank-1 approximation gives a different—and superior, as we argue below—result from a simple linear truncation of the resolvent matrix, which would yield instead

[46] $$\begin{array}{c} K_{s}≃\left(1+\alpha G+O\left(\alpha^{2}\right)\right)1=1+\alpha k\;. \end{array}$$

To make further analytical progress, we appeal to the Law of Large Numbers for large N to further approximate

[47] $$\begin{array}{cc} \sum_{i}k_{i} & \approx N\sum_{k=0}^{\infty }kp\left(k\right)\equiv Nc, \end{array}$$

[48] $$\begin{array}{cc} \sum_{i}k_{i}^{2} & \approx N\sum_{k=0}^{\infty }k^{2}p\left(k\right)\equiv N\bar{k^{2}}\;. \end{array}$$

The relation Eq. **45** allows us to write an approximate formula for the pdf of the Katz centrality for a large network with degree distribution p(k) as

[49] $$\begin{array}{c} P\left(K\right)≃\sum_{k=0}^{\infty }p\left(k\right)\delta \left(K-\frac{\alpha }{1-\alpha \frac{\bar{k^{2}}}{c}}k\right)\;. \end{array}$$

Specializing for instance to a large Erdős-Rényi network with finite mean degree^¶^c—characterized by a Poisson degree distribution $p\left(k\right)=e^{-c}c^{k}/k!$—we see that the centrality distribution is approximated by a Poisson-weighted Dirac comb

[50] $$\begin{array}{c} P\left(K\right)≃\sum_{k=0}^{\infty }e^{-c}\frac{c^{k}}{k!}\delta \left(K-\frac{\alpha }{1-\alpha (1+c)}k\right)\;, \end{array}$$

where we used

[51] $$\begin{array}{cc} \sum_{k=0}^{\infty }k\frac{e^{-c}c^{k}}{k!} & =c, \end{array}$$

[52] $$\begin{array}{cc} \sum_{k=0}^{\infty }k^{2}\frac{e^{-c}c^{k}}{k!} & =c+c^{2}\;. \end{array}$$

**Fig. 11.**
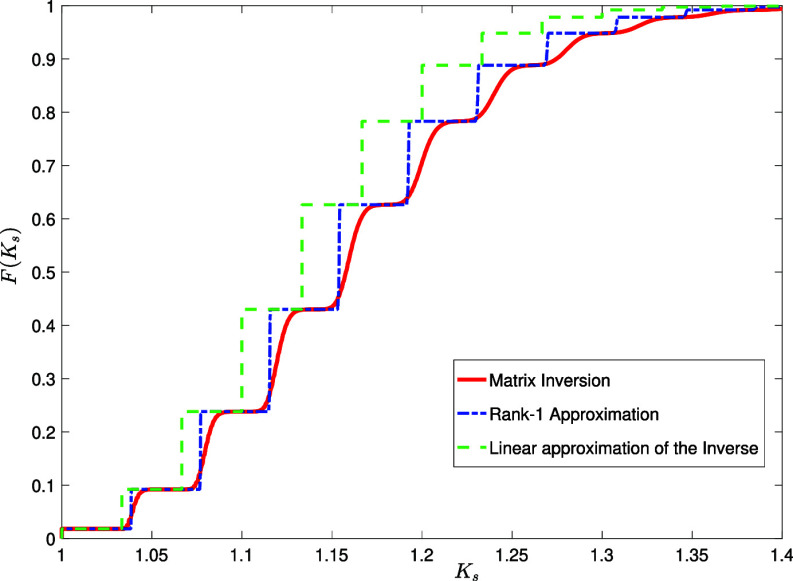
Red line: empirical Cumulative Distribution Function (CDF) $F\left(K_{s}\right)=\int_{0}^{K_{s}}P\left(K^{\prime }\right)dK^{\prime }$ of the shifted Katz centrality $K_{s}$ for an ensemble of 30 randomly generated Erdős-Rényi networks of size N=5,000 with c=4 and α=1/30, using the inversion formula Eq. **5**. Blue dash-dotted line: CDF of the Dirac comb approximate formula Eq. **50** with $K=K_{s}-1$. Green dashed line: CDF of the Dirac comb formula resulting from a simple linear approximation of the resolvent (Eq. **46**).

In Fig. 11, we plot in red the empirical Cumulative Distribution Function (CDF) $F\left(K_{s}\right)=\int_{0}^{K_{s}}P\left(K^{\prime }\right)dK^{\prime }$ (the probability of observing a node with shifted Katz centrality smaller than $K_{s}$) over an ensemble of 30 randomly generated Erdős-Rényi networks of size N=5,000 with c=4 and α=1/30. In blue, we plot the CDF of the theoretical approximate formula Eq. **50**, and in green the CDF of the Dirac comb formula corresponding to a crude linear truncation of the resolvent matrix (Eq. **46**). This corresponds to replacing the term $\delta \left(K-\frac{\alpha }{1-\alpha (1+c)}k\right)$ with $\delta \left(K-\alpha k\right)$ in Eq. **50**.

We observe that the approximate formula Eq. **50** works very well for higher c throughout the full set of allowed values of α (Eq. **2**), whereas for lower c—where the actual distribution has a pronounced multimodality—it correctly reproduces the typical values of the centrality possessed by nodes of degree k (i.e. the location of the k-th peak) and the value of the probability mass under each peak. The “network” effect in a sparse regime therefore essentially amounts to dressing the degree-only information with some noise. Moreover, in Fig. 11, we also show that the CDF of the approximate Dirac comb formula that would result from using a simple linear truncation of the resolvent matrix (Eq. **46**) does not capture the location of the peaks nearly as accurately as our rank-1 approximation, with a clear shift of all values to the left.

## 9. Conclusions and Outlook

In this work, we considered the distribution of the Katz centrality of nodes on single instances and on ensembles of undirected random graphs in the locally tree-like regime, focusing in particular on Erdős-Rényi and Scale Free networks. The Katz centrality of a node is a measure of how important that node is in the context of information flow across the network, as it is a weighted sum of paths of all lengths reaching that node from all other nodes, where longer paths are weighted less by a factor α. Having accurate analytical control over the full distributions in “null models” (with interactions drawn at random with a prescribed distribution that typically preserves the degree sequence/distribution) is important to provide a benchmark to gauge deviations arising in empirical and synthetic data. However, the available analytical results for the full distribution of centrality measures on random networks are surprisingly scarce, which motivates the work we presented here.

The (shifted) Katz centralities of all nodes satisfy a linear system of Eq. **6**, which can be efficiently solved on a single instance of the network using the cavity method (or Gaussian Belief Propagation algorithm). We reviewed in detail the underlying theory in Section 3.

From the single instance solution, it is straightforward to deduce that the probability P(K) of observing a node with centrality K in an ensemble of random networks can be computed from the functional solution of a pair of recursive distributional equations (Eqs. **36** and **37**), which can be efficiently solved using a Population Dynamics algorithm as described in Section 6.

Our results further confirm that the Katz centrality is highly correlated with the degree of nodes, with the k-th peak in the distribution precisely corresponding to the contributions of nodes of degree k to the centrality. The sharply multimodal distribution of the centrality for low c gradually crosses over toward a unimodal distribution as the average degree c increases, with different peaks merging together.

The distribution of centrality across nodes of the same degree in a random network can be further used as a benchmark to identify nodes of an empirical network whose centrality is under- or overexpressed relative to their degree. In the airline routes example we considered, we indeed showed that an airport having few or many connecting routes (its degree) does not tell the full story about its functional role within the airline industry: the “centrality” dimension is also important e.g. to identify smaller airports that nonetheless serve a crucial role in ensuring global connectivity, or conversely very busy ones that only serve local or regional communities, though.

Our approach has wider applications in different fields. For instance, in finance and economics preliminary attempts to determine whether a node would significantly contribute to a shock propagation have been recently made (107). In biology, our approach could be useful to determine relevant sets of genes and proteins (and their functional multiscale role) in different biological processes (108).

Moreover, we have provided an analytical approximation for the centrality distribution, which is based on the rank-1 projection proposed in ref. 65 and works well for not-too-sparse graphs. If the graphs are very sparse, the approximation is anyway able to capture the location and mass of each peak in a more accurate way than a simple linear truncation of the resolvent matrix.

It will be interesting to modify the treatment presented here to deal with the case of networks with correlated degrees, as well as directed networks for which the GaBP/cavity solution of a linear system Eq. **7** on a tree structure requires some changes (109). Extending the analysis to nonsymmetric adjacency matrices would allow us to deal for instance with the distribution of PageRank in random networks, a topic that has received some attention in the mathematical literature lately in the context of the so-called “power-law hypothesis” described in *Introduction*.

## Supplementary Material

Appendix 01 (PDF)

## Data Availability

Previously published data were used for this work (70).

## Footnotes

^*^While power-law networks with exponent less than 3 have finite loops (66), the tree-like approximation appears to work well also on these structures (6).

^†^We have ${\left(K_{s}\right)}_{i}\geq 0$ from ref. 81. Then, ${\left(K_{s}\right)}_{i}=1+\alpha {\left(GK_{s}\right)}_{i}$ from Eq. **6**. Since G has nonnegative entries and α is nonnegative, the claim easily follows.

^‡^The theory developed here for configuration model ensembles with no degree–degree correlation can be extended, allowing for a more general probability $P(k^{\prime }|k)$ that a node of degree k is connected to a node of degree $k^{\prime }$. In this case, the distribution π(μ,V) needs to be made k-dependent, $\pi_{k}\left(\mu ,V\right)$ (103). The case of no degree–degree correlation is recovered by noting that in this case $P(k^{\prime }|k)$ is independent of k. Therefore, $P(k^{\prime }|k)$ reduces to the probability that an edge points to a node of degree $k^{\prime }$, which can be defined as the ratio between the number of edges pointing to nodes of degree $k^{\prime }$, $k^{\prime }p\left(k^{\prime }\right)$, and the number of edges pointing to nodes of any degree, i.e. the sum $\sum_{k^{\prime }}k^{\prime }p\left(k^{\prime }\right)=c$.

^§^The value of α should ideally be chosen in such a way that for *all* instances G of graphs in the ensemble, the matrix (1−αG) is positive definite - or that at least violations of this requirement are “sufficiently rare.” While we are not aware of any sound mathematical treatment of how small the value α should be to ensure that violations of positive definiteness occur less frequently than a given tolerance, in practice the values of α we employ are sufficiently small that no practical issues have arisen in our simulations.

^¶^On scale-free networks with exponent γ, the second moment diverges with N. If we consider the structural cutoff $k_{\;\text{max}}∼N^{1/2}$, we have that $\left\langle k^{2}\right\rangle ∼N^{(3-\gamma )/2}$. This implies that α should go to zero as N increases for Eq. **49** to be meaningful. A similar conclusion can be reached from the condition in Eq. **2** using the results for the maximum eigenvalue of networks generated with the configuration model reported in ref. 106.

## References

[1] R. Albert, H. Jeong, A.-L. Barabási, Error and attack tolerance of complex networks. Nature **406**, 378–382 (2000). 10935628 10.1038/35019019

[2] D. S. Callaway, M. E. J. Newman, S. H. Strogatz, D. J. Watts, Network robustness and fragility: Percolation on random graphs. Phys. Rev. Lett. **85**, 5468–5471 (2000). 11136023 10.1103/PhysRevLett.85.5468

[3] H. Jeong, S. P. Mason, A.-L. Barabási, Z. N. Oltvai, Lethality and centrality in protein networks. Nature **411**, 41–42 (2001). 11333967 10.1038/35075138

[4] M. De Domenico, A. Solé-Ribalta, S. Gómez, A. Arenas, Navigability of interconnected networks under random failures. Proc. Natl. Acad. Sci. U.S.A. **111**, 8351–8356 (2014). 24912174 10.1073/pnas.1318469111 PMC4060702

[5] S. Boccaletti, V. Latora, Y. Moreno, M. Chavez, D.-U. Hwang, Complex networks: Structure and dynamics. Phys. Rep. **424**, 175–308 (2006).

[6] A. V. Goltsev, S. N. Dorogovtsev, J. G. Oliveira, J. F. F. Mendes, Localization and spreading of diseases in complex networks. Phys. Rev. Lett. **109**, 128702 (2012). 23006000 10.1103/PhysRevLett.109.128702

[7] J. Gao, B. Barzel, A.-L. Barabási, Universal resilience patterns in complex networks. Nature **530**, 307–312 (2016). 26887493 10.1038/nature16948

[8] N. Crua Asensio, E. Muñoz Giner, N. S. de Groot, M. Torrent Burgas, Centrality in the host-pathogen interactome is associated with pathogen fitness during infection. Nat. Commun. **8**, 14092 (2017). 28090086 10.1038/ncomms14092 PMC5241799

[9] H. Farooq, Y. Chen, T. T. Georgiou, A. Tannenbaum, C. Lenglet, Network curvature as a hallmark of brain structural connectivity. Nat. Commun. **10**, 4937 (2019). 31666510 10.1038/s41467-019-12915-x PMC6821808

[10] D. Guilbeault, D. Centola, Topological measures for identifying and predicting the spread of complex contagions. Nat. Commun. **12**, 4430 (2021). 34285206 10.1038/s41467-021-24704-6 PMC8292353

[11] D. Bucur, P. Holme, Beyond ranking nodes: Predicting epidemic outbreak sizes by network centralities. PLoS Comput. Biol. **16**, e1008052 (2020). 32697781 10.1371/journal.pcbi.1008052 PMC7398553

[12] M. Bardoscia , The physics of financial networks. Nat. Rev. Phys. **3**, 490–507 (2021).

[13] D. Chen, L. Lü, M. S. Shang, Y. C. Zhang, T. Zhou, Identifying influential nodes in complex networks. Phys. A **391**, 1777–1787 (2012).

[14] G. Ghoshal, A.-L. Barabási, Ranking stability and super-stable nodes in complex networks. Nat. Commun. **2**, 1–7 (2011). 10.1038/ncomms1396 21772265

[15] R. Guimerà, S. Mossa, A. Turtschi, L. A. N. Amaral, The worldwide air transportation network: Anomalous centrality, community structure, and cities’ global roles. Proc. Natl. Acad. Sci. U.S.A. **102**, 7794–7799 (2005). 15911778 10.1073/pnas.0407994102 PMC1142352

[16] Z. Wu, L. A. Braunstein, S. Havlin, H. E. Stanley, Transport in weighted networks: Partition into superhighways and roads. Phys. Rev. Lett. **96**, 148702 (2006). 16712129 10.1103/PhysRevLett.96.148702

[17] G. Brown, M. Carlyle, J. Salmerón, K. Wood, Defending critical infrastructure. Interfaces **36**, 530–544 (2006).

[18] R. Carvalho , Robustness of trans-european gas networks. Phys. Rev. E **80**, 016106 (2009). 10.1103/PhysRevE.80.016106 19658773

[19] Y. Duan, F. Lu, Robustness of city road networks at different granularities. Phys. A Stat. Mech. Appl. **411**, 21–30 (2014).

[20] S. Brin, L. Page, The anatomy of a large-scale hypertextual Web search engine. Comput. Netw. ISDN Syst. **30**, 107–117 (1998).

[21] L. Page, S. Brin, R. Motwani, T. Winograd, The PageRank Citation Ranking: Bringing Order to the Web (Stanford InfoLab, 1999).

[22] M. Kitsak , Identification of influential spreaders in complex networks. Nat. Phys. **6**, 888–893 (2010).

[23] M. Salathé , A high-resolution human contact network for infectious disease transmission. Proc. Natl. Acad. Sci. U.S.A. **107**, 22020–22025 (2010). 21149721 10.1073/pnas.1009094108 PMC3009790

[24] R. Pung, J. A. Firth, Spurgin, Singapore CruiseSafe working group, and CMMID COVID-19 working group, Using high-resolution contact networks to evaluate SARS-CoV-2 transmission and control in large-scale multi-day events. Nat. Commun. **13**, 1956 (2022). 35414056 10.1038/s41467-022-29522-y PMC9005731

[25] R. V. Solé, M. Rosas-Casals, B. Corominas-Murtra, S. Valverde, Robustness of the european power grids under intentional attack. Phys. Rev. E **77**, 026102 (2008). 10.1103/PhysRevE.77.026102 18352085

[26] J. C. Doyle , The “robust yet fragile’’ nature of the internet. Proc. Natl. Acad. Sci. U.S.A. **102**, 14497–14502 (2005). 16204384 10.1073/pnas.0501426102 PMC1240072

[27] S. M. Rinaldi, J. P. Peerenboom, T. K. Kelly, Identifying, understanding, and analyzing critical infrastructure interdependencies. IEEE control Syst. Mag. **21**, 11–25 (2001).

[28] R. Cohen, K. Erez, D. Ben Avraham, S. Havlin, Resilience of the internet to random breakdowns. Phys. Rev. Lett. **85**, 4626–4628 (2000). 11082612 10.1103/PhysRevLett.85.4626

[29] B. Schäfer, D. Witthaut, M. Timme, V. Latora, Dynamically induced cascading failures in power grids. Nat. Commun. **9**, 1975 (2018). 29773793 10.1038/s41467-018-04287-5 PMC5958123

[30] P. Bonacich, Factoring and weighting approaches to status scores and clique identification. J. Math. Sociol. **2**, 113–120 (1972).

[31] L. Katz, A new status index derived from sociometric analysis. Psychometrika **18**, 39–43 (1953).

[32] L. C. Freeman, A set of measures of centrality based on betweenness. Sociometry **40**, 35–41 (1977).

[33] M. E. J. Newman, A measure of betweenness centrality based on random walks. Soc. Netw. **27**, 39–54 (2005).

[34] E. Estrada, The Structure of Complex Networks (Oxford University Press, Oxford, 2011).

[35] M. Engsig , DomiRank Centrality reveals structural fragility of complex networks via node dominance. Nat. Commun. **15**, 56 (2024). 38167342 10.1038/s41467-023-44257-0 PMC10761873

[36] U. Brandes, D. Fleischer, “Centrality measures based on current flow” in STACS 2005: Lecture Notes in Computer Science, V. Diekert, B. Durand, Eds. (Springer, Berlin, Heidelberg, 2005).

[37] A. Ghavasieh, M. Stella, J. Biamonte, M. De Domenico, Unraveling the effects of multiscale network entanglement on empirical systems. Commun. Phys. **4**, 129 (2021).

[38] G. F. de Arruda , Role of centrality for the identification of influential spreaders in complex networks. Phys. Rev. E **90**, 032812 (2014). 10.1103/PhysRevE.90.032812 25314487

[39] F. Bloch, M. O. Jackson, P. Tebaldi, Centrality measures in networks. Soc. Choice Welf. **61**, 413–453 (2023).

[40] A. Saxena, S. Iyengar, Centrality measures in complex networks: A survey. arXiv [Preprint]. (2020). http://arxiv.org/abs/2011.07190 (Accessed 29 June 2024).

[41] Z. Wan, Y. Mahajan, B. W. Kang, T. J. Moore, J.-H. Cho, A survey on centrality metrics and their network resilience analysis. IEEE Access **9**, 104773–104819 (2021).

[42] P. Boldi, S. Vigna, Axioms for centrality. Internet Math. **10**, 222–262 (2014).

[43] N. Fraiman, T.-C. Lin, M. Olvera-Cravioto, Stochastic recursions on directed random graphs. Stoch. Process. Appl. **166**, 104055 (2023).

[44] A. Garavaglia, R. van der Hofstad, N. Litvak, Local weak convergence for PageRank. Ann. Appl. Probab. **30**, 40–79 (2020).

[45] P. R. Jelenković, M. Olvera-Cravioto, Information ranking and power laws on trees. Adv. Appl. Probab. **42**, 1057–1093 (2010).

[46] J. Lee, M. Olvera-Cravioto, PageRank on inhomogeneous random digraphs. Stoch. Process. Appl. **130**, 1–57 (2020).

[47] M. Olvera-Cravioto, Tail behavior of solutions of linear recursions on trees. Stoch. Process. Appl. **122**, 1777–1807 (2012).

[48] M. Olvera-Cravioto, PageRank’s behavior under degree correlations. Ann. Appl. Probab. **31**, 1403–1442 (2021).

[49] K. Avrachenkov, D. Lebedev, PageRank of scale-free growing networks. Internet Math. **3**, 207–231 (2006).

[50] Y. Volkovich, N. Litvak, Asymptotic analysis for personalized Web search. Adv. Appl. Probab. **42**, 577–604 (2010).

[51] S. Banerjee, M. Olvera-Cravioto, PageRank asymptotics on directed preferential attachment networks. Ann. Appl. Probab. **32**, 3060 (2022).

[52] N. Litvak, W. R. W. Scheinhardt, Y. Volkovich, In-degree and PageRank: Why do they follow similar power laws? Internet Math. **4**, 175–198 (2011).

[53] G. Pandurangan, P. Raghavan, E. Upfal, Using PageRank to Characterize Web Structure. Internet Math. **3**, 1–20 (2006).

[54] D. Donato, L. Laura, S. Leonardi, S. Millozi, Large scale properties of the webgraph. Eur. Phys. J. **38**, 239–243 (2004).

[55] S. Fortunato, M. Boguñá, A. Flammini, and F. Menczer, “Approximating pagerank from in-degree” in *Algorithms and Models for the Web-Graph. WAW 2006*, W. Aiello, A. Broder, J. Janssen, E. Milios, Eds. (Lecture Notes in Computer Science, Springer, Berlin, Heidelberg, 2008), vol. 4936.

[56] L. Becchetti and C. Castillo, “The distribution of PageRank follows a power-law only for particular values of the damping factor” in *Proceedings of the 15th International Conference on World Wide Web* (New York: ACM Press, 2006), pp. 941–942.

[57] P.-E. Lu, C.-S. Chang, D.-S. Lee, C.-C. Huang, Centrality analysis in *d*-regular directed acyclic random networks and its applications in top-*k* recommendations. IEEE Trans. Comput. Soc. Syst. **6**, 968–980 (2019).

[58] C. Durón, “The distribution of betweenness centrality in exponential random graph models,” PhD thesis, Pomona College, Claremont (CA) (2019). https://pages.pomona.edu/~jsh04747/Student%20Theses/christina_duron_2019.pdf.

[59] K. Durant, S. Wagner, On the distribution of betweenness centrality in random trees. Theor. Comput. Sci. **699**, 33–52 (2017).

[60] M. Paton, K. Akartunali, D. J. Higham, Centrality analysis for modified lattices. SIAM J. Matrix Anal. Appl. **38**, 1055 (2017).

[61] K. Avrachenkov, A. Kadavankandy, L. Ostroumova Prokhorenkova, A. Raigorodskii, “PageRank in undirected random graphs” in *Algorithms and Models for the Web Graph. WAW. 2015. Lecture Notes in Computer Science*, D. Gleich, J. Komjáthy, N. Litvak, Eds. (Springer, Cham, 2015), vol. 9479, p. 2015.

[62] N. Perra, S. Fortunato, Spectral centrality measures in complex networks. Phys. Rev. E **78**, 036107 (2008). 10.1103/PhysRevE.78.036107 18851105

[63] P. Crucitti, V. Latora, S. Porta, Centrality measures in spatial networks of urban streets. Phys. Rev. E **73**, 036125 (2006). 10.1103/PhysRevE.73.036125 16605616

[64] A. Kirkley, H. Barbosa, M. Barthelemy, G. Ghoshal, From the betweenness centrality in street networks to structural invariants in random planar graphs. Nat. Commun. **9**, 2501 (2018). 29950619 10.1038/s41467-018-04978-z PMC6021391

[65] S. Bartolucci, F. Caccioli, F. Caravelli, P. Vivo, Ranking influential nodes in networks from aggregate local information. Phys. Rev. Res. **5**, 033123 (2023).

[66] G. Bianconi, M. Marsili, Loops of any size and Hamilton cycles in random scale-free networks. J. Stat. Mech. **2005**, P06005 (2005).

[67] Y. Weiss, W. T. Freeman, Correctness of belief propagation in gaussian graphical models of arbitrary topology. Neural Comput. **13**, 2173–2200 (2001). 11570995 10.1162/089976601750541769

[68] O. Shental, P. H. Siegel, J. K. Wolf, D. Bickson, D. Dolev, “Gaussian belief propagation solver for systems of linear equations,” in *2008 IEEE International Symposium on Information Theory* (Toronto, ON, Canada, 2008), pp. 1863–1867.

[69] D. Bickson, Gaussian belief propagation: Theory and application. arXiv [Preprint] (2009). http://arxiv.org/abs/0811.2518 (Accessed 29 June 2024).

[70] T. Woebkenberg, Data from “Global air transportation network: Airports, airlines, and routes.” Kaggle. https://www.kaggle.com/datasets/thedevastator/global-air-transportation-network-mapping-the-wo/data. Accessed 29 June 2024.

[71] J. Leskovec, J. Mcauley, “Learning to discover social circles in ego networks” in *Advances in Neural Information Processing Systems*, F. Pereira, C. J. Burges, L. Bottou, K. Q. Weinberger (Curran Associates, Inc., 2012), vol. 25.

[72] J. Leskovec, J. Kleinberg, C. Faloutsos, Graph evolution: Densification and shrinking diameters. ACM Trans. Knowl. Discov. Data **1**, 2–es (2007).

[73] S. Bartolucci, F. Caccioli, F. Caravelli, P. Vivo, “Spectrally gapped’’ random walks on networks: A mean first passage time formula. SciPost Phys. **11**, 088 (2021).

[74] S. Bartolucci, F. Caccioli, F. Caravelli, P. Vivo, Upstreamness and downstreamness in input-output analysis from local and aggregate information. arXiv [Preprint] (2024). http://arxiv.org/abs/2009.06350v4 (Accessed 29 June 2024). 10.1038/s41598-025-86380-6 PMC11751120 39837945

[75] T. S. Evans, B. Chen, Linking the network centrality measures closeness and degree. Commun. Phys. **5**, 172 (2022).

[76] S. Oldham , Consistency and differences between centrality measures across distinct classes of networks. PLoS One **14**, e0220061 (2019). 31348798 10.1371/journal.pone.0220061 PMC6660088

[77] T. W. Valente, K. Coronges, C. Lakon, E. Costenbader, How correlated are network centrality measures? Connect. (Tor. Ont) **28**, 16–26 (2008). PMC2875682 20505784

[78] C. Li, Q. Li, P. Van Mieghem, H. E. Stanley, H. Wang, Correlation between centrality metrics and their application to the opinion model. Eur. Phys. J. B **88**, 65 (2015).

[79] M. Aprahamian, D. J. Higham, N. J. Higham, Matching exponential-based and resolvent-based centrality measures. J. Complex Netw. **4**, 157–176 (2016).

[80] M. Benzi, C. Klymko, On the limiting behavior of parameter-dependent network centrality measures. SIAM J. Matrix Anal. Appl. **36**, 686–706 (2015).

[81] B. Peterson, M. Olinick, Leontief models, Markov chains, substochastic matrices, and positive solutions of matrix equations. *Math. Model.* **3**, 221–239 (1982).

[82] D. Bickson, D. Malkhi, A unifying framework of rating users and data items in peer-to-peer and social networks. Peer Peer Netw. Appl. **1**, 93–103 (2008).

[83] Y. Saad, “Iterative methods for sparse linear systems” in *Society for Industrial and Applied Mathematics* (ed. 2, 2003).

[84] E. Nathan, G. Sanders, J. Fairbanks, V. E. Henson, D. A. Bader, Graph ranking guarantees for numerical approximations to Katz centrality. Proc. Comput. Sci. **108**, 68–78 (2017).

[85] D. Bickson, Y. Tock, A. Zymnis, S. P. Boyd, D. Dolev, “Distributed large scale network utility maximization” in *2009 IEEE International Symposium on Information Theory* (Seoul, South Korea, 2009), pp. 829–833.

[86] D. Bickson, O. Shental, P. H. Siegel, J. K. Wolf, D. Dolev, “Linear detection via belief propagation” in *45th Annual Allerton Conference on Communication, Control, and Computing* (University of Illinois, Monticello, Illinois, 2007), vol. 2, pp. 1289.

[87] M. Mézard, G. Parisi, M. Virasoro, Spin Glass Theory and Beyond: An Introduction to the Replica Method and Its Applications (World Scientific Publishing Company, 1987), vol. 9.

[88] T. Rogers, I. Pérez Castillo, R. Kühn, K. Takeda, Cavity approach to the spectral density of sparse symmetric random matrices. Phys. Rev. E **78**, 031116 (2008). 10.1103/PhysRevE.78.031116 18851002

[89] P. Cizeau, J.-P. Bouchaud, Theory of Lévy matrices. Phys. Rev. E **50**, 1810 (1994). 10.1103/physreve.50.1810 9962183

[90] L. Zdeborová, F. Krzakala, Statistical physics of inference: Thresholds and algorithms. Adv. Phys. **65**, 453–552 (2016).

[91] J. Barbier, F. Krzakala, N. Macris, L. Miolane, L. Zdeborová, Optimal errors and phase transitions in high-dimensional generalized linear models. Proc. Natl. Acad. Sci. U.S.A. **116**, 5451–5460 (2019). 30824595 10.1073/pnas.1802705116 PMC6431156

[92] D. L. Donoho, A. Maleki, A. Montanari, Message-passing algorithms for compressed sensing. Proc. Natl. Acad. Sci. U.S.A. **106**, 18914–18919 (2009). 19858495 10.1073/pnas.0909892106 PMC2767368

[93] J. K. Johnson, D. M. Malioutov, A. S. Willsky, “Walk-sum interpretation and analysis of Gaussian belief propagation” in *Advances in Neural Information Processing Systems 18*, Y. Weiss, B. Schölkopf, J. Platt, Eds. (MIT Press, Cambridge, MA, 2006), pp. 579–586.

[94] V. A. R. Susca, P. Vivo, R. Kühn, Cavity and replica methods for the spectral density of sparse symmetric random matrices. SciPost Phys. Lect. Notes, 33 (2021).

[95] C. Y. Lee, Correlations among centrality measures in complex networks. arXiv [Preprint]. (2006). 10.48550/arXiv.physics/0605220 (Accessed 29 June 2024).

[96] R. Kühn, J. Van Mourik, M. Weigt, A. Zippelius, Finitely coordinated models for low-temperature phases of amorphous systems. J. Phys. A Math. Theor. **40**, 9227 (2007).

[97] M. Mézard, G. Parisi, The Bethe lattice spin glass revisited. Eur. Phys. J. B **20**, 217–233 (2001).

[98] F. Krzakala , Statistical Physics, Optimization, Inference, and Message-Passing Algorithms (Oxford University Press, 2016).

[99] R. Kühn, Spectra of sparse random matrices. J. Phys. A Math. Theor. **41**, 295002 (2008).

[100] V. A. R. Susca, P. Vivo, R. Kühn, Top eigenpair statistics for weighted sparse graphs. J. Phys. A Math. Theor. **52**, 485002 (2019).

[101] V. A. R. Susca, P. Vivo, R. Kühn, Second largest eigenpair statistics for sparse graphs. J. Phys. A Math. Theor. **54**, 015004 (2021).

[102] R. Kühn, T. Rogers, Heterogeneous micro-structure of percolation in sparse networks. Europhys. Lett. **118**, 68003 (2017).

[103] A. Vázquez, M. Weigt, Computational complexity arising from degree correlations in networks. Phys. Rev. E **67**, 027101 (2003). 10.1103/PhysRevE.67.027101 12636856

[104] M. Catanzaro, M. Boguñá, R. Pastor-Satorras, Generation of uncorrelated random scale-free networks. Phys. Rev. E **71**, 027103 (2005). 10.1103/PhysRevE.71.027103 15783457

[105] J. Sherman, W. J. Morrison, Adjustment of an inverse matrix corresponding to a change in one element of a given matrix. Ann. Math. Stat. **21**, 124–127 (1950).

[106] P. Dionigi, D. Garlaschelli, R. S. Hazra, F. D. Hollander, Largest eigenvalue of the configuration model and breaking of ensemble equivalence. arXiv [Preprint] (2023). http://arxiv.org/abs/2312.07812 (Accessed 29 June 2024).

[107] A. Sadeghi, Z. Feinstein, Statistical validation of contagion centrality in financial networks. arXiv [Preprint] (2024). http://arxiv.org/abs/2404.14337 (Accessed 29 June 2024).

[108] Z. Gu, J. Liu, K. Cao, J. Zhang, J. Wang, Centrality-based pathway enrichment: A systematic approach for finding significant pathways dominated by key genes. BMC Syst. Biol. **6**, 1–13 (2012). 22672776 10.1186/1752-0509-6-56 PMC3443660

[109] V. Fanaskov, Gaussian belief propagation solvers for nonsymmetric systems of linear equations. SIAM J. Sci. Comput. **44**, A77–A102 (2022).

