# Supplementary material for: Distribution of centrality measures on undirected random networks via the cavity method
Source: Proc Natl Acad Sci U S A. 2024 Sep 25;121(40):e2403682121. doi: 10.1073/pnas.2403682121 (PMC11459148; doi:10.1073/pnas.2403682121)
Supplement: Supplementary file 1 — Appendix 01 (PDF) [file pnas.2403682121.sapp.pdf]

Supporting Information for

Distribution of centrality measures on undirected random networks via cavity method

Silvia Bartolucci, Francesco Caravelli, Fabio Caccioli, Pierpaolo Vivo

Corresponding Pierpaolo Vivo
E-mail: pierpaolo.vivo@kcl.ac.uk

This PDF file includes:

- Supporting text
- Figs. S1 to S10
- Table S1
- SI References

Supporting Information Text

A. Random Regular Graphs. As a simple further check of the formalism, we may specialize the cavity equations (31-34) of the main text to the case of a random regular graph having all nodes with the same degree, $p(k) = \delta_{k,c}$. The Katz centrality of all nodes is the same, and given by

$$K_i = \frac{1}{1-\alpha c} - 1 \qquad \forall i \;, \tag{1}$$

(see Lemma 3.1 in (1)).

The set of recursive equations above specializes to

$$V = \frac{1}{1-\alpha^2(c-1)V} \tag{2}$$

$$\mu = V\left(1+\alpha(c-1)\mu\right) \tag{3}$$

$$\tilde{V} = \frac{1}{1-\alpha^2 cV} \tag{4}$$

$$\tilde{\mu} = \tilde{V}\left(1+\alpha c\mu\right) \;, \tag{5}$$

where we imposed that all cavity fields take up a single value (μ and V) on every edge, and similarly for the marginal fields ($\tilde{\mu}$ and $\tilde{V}$). The equations above can be easily solved, and the value of $\tilde{\mu} = 1/(1-\alpha c)$. It follows therefore from Eq. (35) of the main text that the Katz centrality of nodes in a random regular graph indeed comes out as Eq. (1).

B. Approximate expression for the conditional variance σ_k^2. For the case of a graph with Poissonian degree distribution with mean c, and under some further conditions on α and c, it is possible to seek an approximate analytical solution of the recursive distributional equation (36) of the main text for $\pi(\mu, V)$, which in turn makes it possible to compute the variance σ_k^2 of the distribution $P(K_s|k)$ of the shifted Katz centrality, conditioned on nodes of degree k.

Assume the following ansatz for $\pi(\mu, V)$

$$\pi(\mu, V) = \frac{e^{-\frac{(\mu-\bar{\mu})^2}{2\Delta}}}{\sqrt{2\pi\Delta}}\delta(V-\bar{V}) \;, \tag{6}$$

where fluctuations of V are neglected, and fluctuations of μ are assumed to be Gaussian, with mean $\bar{\mu}$ and variance Δ to be determined self-consistently. Assume also that c is sufficiently large that the Poissonian degree distribution $p(k) = e^{-c}c^k/k!$ can be faithfully approximated by a normal distribution with mean c and variance c

$$\sum_{k\geq 1} p(k)\frac{k}{c}f(k) \to \int_{-\infty}^{\infty}\frac{dk}{\sqrt{2\pi c}}e^{-\frac{(k-c)^2}{2c}}\frac{k}{c}f(k) \tag{7}$$

for a test function $f(k)$.

Then, we can cast the right hand side of (36) of the main text in the following approximate form

$$\pi(\mu, V=\bar{V}) \approx \int_{-\infty}^{\infty}\frac{d\phi}{2\pi}e^{\mathrm{i}\phi\mu}\int_{-\infty}^{\infty}\frac{dk}{\sqrt{2\pi c}}e^{-\frac{(k-c)^2}{2c}}\frac{k}{c}\int d\mu_1\cdots d\mu_{k-1}\left[\prod_{j=1}^{k-1}\frac{e^{-\frac{-(\mu_j-\bar{\mu})^2}{2\Delta}}}{\sqrt{2\pi\Delta}}\right]\exp\left[-\mathrm{i}\phi\bar{V}\left(1+\alpha\sum_{\ell=1}^{k-1}\mu_\ell\right)\right] \;, \tag{8}$$

where we have used the Fourier representation of Dirac delta

$$\delta(x) = \int\frac{d\phi}{2\pi}e^{\mathrm{i}\phi x} \;, \tag{9}$$

and the value $\bar{V}$ is determined from the condition in Eq. (2) assuming that we can approximate k with its most likely value $k = c$,

$$\bar{V} = \frac{1}{1-\alpha^2(c-1)\bar{V}} \;. \tag{10}$$

The $(k-1)$-fold integral can now be performed to yield

$$\pi(\mu, V=\bar{V}) \approx \int_{-\infty}^{\infty}\frac{d\phi}{2\pi}e^{\mathrm{i}\phi(\mu-\bar{V})}\int_{-\infty}^{\infty}\frac{dk}{\sqrt{2\pi c}}e^{-\frac{(k-c)^2}{2c}}\frac{k}{c}\left[\int_{-\infty}^{\infty}\frac{d\mu}{\sqrt{2\pi\Delta}}e^{-\frac{(\mu-\bar{\mu})^2}{2\Delta}-\mathrm{i}\phi\bar{V}\alpha\mu}\right]^{k-1} \;. \tag{11}$$

The μ-integral can be performed using

$$\int_{-\infty}^{\infty}d\mu\; e^{-A\mu^2-\mathrm{i}B\mu} = \sqrt{\frac{\pi}{A}}\exp\left[-\frac{B^2}{4A}\right] \;, \tag{12}$$

with

$$A = \frac{1}{2\Delta} \tag{13}$$

$$B = \phi \bar{V} \alpha + \mathrm{i}\frac{\bar{\mu}}{\Delta} \ , \tag{14}$$

to yield

$$\left[\int_{-\infty}^{\infty} \frac{d\mu}{\sqrt{2\pi\Delta}} e^{-\frac{(\mu-\bar{\mu})^2}{2\Delta} - \mathrm{i}\phi\bar{V}\alpha\mu}\right]^{k-1} = \exp\left[-\frac{\bar{\mu}^2}{2\Delta}(k-1) - (k-1)\frac{\Delta}{2}\left(\phi\bar{V}\alpha + \mathrm{i}\frac{\bar{\mu}}{\Delta}\right)^2\right] \ . \tag{15}$$

Collecting terms, we can write

$$\pi(\mu, V = \bar{V}) \approx \frac{e^{-c/2}}{c\sqrt{2\pi c}} \int_{-\infty}^{\infty} \frac{d\phi}{2\pi} \exp\left[\mathrm{i}\phi(\mu - \bar{V}) + \frac{\Delta}{2}\bar{V}^2\alpha^2\phi^2 + \mathrm{i}\bar{V}\alpha\bar{\mu}\phi\right] \int_{-\infty}^{\infty} dk\ k\ e^{-\frac{1}{2c}k^2 - \mathrm{i}\hat{B}k} \ , \tag{16}$$

where

$$\hat{B} = \mathrm{i} + \phi\bar{V}\alpha\bar{\mu} - \mathrm{i}\frac{\Delta}{2}\bar{V}^2\alpha^2\phi^2 \ . \tag{17}$$

The k-integral can be performed to yield

$$\int_{-\infty}^{\infty} dk\ k\ e^{-\frac{1}{2c}k^2 - \mathrm{i}\hat{B}k} = -\mathrm{i}\sqrt{2\pi}\hat{B}c^{3/2}e^{-\frac{c}{2}\hat{B}^2} \ . \tag{18}$$

Squaring the exponent in Eq. (18) would give

$$-\frac{c}{2}\hat{B}^2 = \frac{c}{2} - \mathrm{i}\mathcal{A}c\phi - \phi^2\left(\frac{c}{2}\mathcal{A}^2 + \mathcal{B}c\right) + \mathrm{i}\mathcal{A}\mathcal{B}c\phi^3 + \frac{1}{2}\mathcal{B}^2c\phi^4 \ , \tag{19}$$

with

$$\mathcal{A} = \bar{V}\alpha\bar{\mu} \tag{20}$$

$$\mathcal{B} = \frac{\Delta}{2}\bar{V}^2\alpha^2 \ . \tag{21}$$

Clearly, the cubic and quartic ϕ-terms in Eq. (19) (as well as the linear and quadratic terms in Eq. (17)) are problematic in view of performing the final ϕ-integration, which would never result in a Gaussian dependence of π on μ as posited in the ansatz Eq. (17). Therefore, we shall further assume that $\alpha \ll 1$ and retain all terms in Eq. (19) up to $\mathcal{O}(\alpha^2)$. This way, the terms $\mathcal{A} \ll 1$ and $\mathcal{B} \ll 1$, and ignoring normalization constants that can be restored at the end, we get

$$\pi(\mu, V = \bar{V}) \approx \int_{-\infty}^{\infty} d\phi\ e^{-D\phi^2 - \mathrm{i}E\phi} \ , \tag{22}$$

with

$$D = \frac{\bar{V}^2\alpha^2}{2}(c\bar{\mu}^2 + \Delta c - \Delta) \tag{23}$$

$$E = -(\mu - \bar{V} + \bar{V}\alpha\bar{\mu}(1-c)) \ , \tag{24}$$

which in turn provides the further condition

$$c\bar{\mu}^2 + \Delta c - \Delta > 0 \tag{25}$$

to ensure convergence.

Using again the Gaussian integration formula Eq. (12), we finally get (ignoring normalization constants)

$$\pi(\mu, V = \bar{V}) \approx \exp\left[-\frac{(\mu - [\bar{V} - \bar{V}\alpha\bar{\mu}(1-c)])^2}{2\bar{V}^2\alpha^2(c\bar{\mu}^2 + \Delta c - \Delta)}\right] \ , \tag{26}$$

which allows us to appeal to the Gaussian ansatz Eq. (6) for π to derive the following set of self-consistency equations for $\bar{\mu}$, $\bar{V}$, and Δ

$$\bar{\mu} = \bar{V} - \bar{V}\alpha\bar{\mu}(1-c) \tag{27}$$

$$\Delta = \bar{V}^2\alpha^2(c\bar{\mu}^2 + \Delta c - \Delta) \tag{28}$$

$$\bar{V} = \frac{1}{1 - \alpha^2(c-1)\bar{V}} \ . \tag{29}$$

The system of equations has two solutions, however one of them is unphysical, yielding to negative variances. Keeping only the physical solution depending on α and c (which is slightly cumbersome, but otherwise fully explicit), we can now proceed and insert the ansatz for π into the k-fold integral (39) of the main text that defines $P(K_s|k)$. This way we may evaluate σ_k^2 as

$$\sigma_k^2 = \langle K_s^2\rangle_k - (\langle K_s\rangle_k)^2 \tag{30}$$

where

$$\langle K_s^2\rangle_k = \int dK_s\ K_s^2 P(K_s|k) = \int \{d\pi\}_k \left(\frac{1}{1-\alpha^2\sum_{\ell=1}^k V_\ell}\right)^2 \left(1+\alpha\sum_{\ell=1}^k \mu_\ell\right)^2 \tag{31}$$

$$\approx \left(\frac{1}{1-\alpha^2 k\bar{V}}\right)^2 \int d\mu_1\cdots d\mu_k \left[\left(\prod_{j=1}^k \frac{e^{-\frac{-(\mu_j-\bar{\mu})^2}{2\Delta}}}{\sqrt{2\pi\Delta}}\right)\left(1+\alpha^2\left(\sum_\ell \mu_\ell\right)^2 + 2\alpha\sum_\ell \mu_\ell\right)\right] \tag{32}$$

$$\approx \left(\frac{1}{1-\alpha^2 k\bar{V}}\right)^2 \left[1+\alpha^2\left(k(\Delta+\bar{\mu}^2)+k(k-1)\bar{\mu}^2\right)+2\alpha k\bar{\mu}\right] \tag{33}$$

and we used

$$\left(\sum_\ell \mu_\ell\right)^2 = \sum_{\ell,m}\mu_\ell\mu_m = \sum_\ell \mu_\ell^2 + \sum_{\ell\neq m}\mu_\ell\mu_m \tag{34}$$

and the integral defining moments of the Gaussian probability density

$$\int d\mu\ \frac{e^{-\frac{-(\mu-\bar{\mu})^2}{2\Delta}}}{\sqrt{2\pi\Delta}}\mu = \bar{\mu} \tag{35}$$

$$\int d\mu\ \frac{e^{-\frac{-(\mu-\bar{\mu})^2}{2\Delta}}}{\sqrt{2\pi\Delta}}\mu^2 = \Delta + \bar{\mu}^2\ . \tag{36}$$

Similarly

$$\langle K_s\rangle_k = \int dK_s\ K_s P(K_s|k) = \int \{d\pi\}_k \left(\frac{1}{1-\alpha^2\sum_{\ell=1}^k V_\ell}\right)\left(1+\alpha\sum_{\ell=1}^k \mu_\ell\right) \tag{37}$$

$$\approx \left(\frac{1}{1-\alpha^2 k\bar{V}}\right)\int d\mu_1\cdots d\mu_k \left[\left(\prod_{j=1}^k \frac{e^{-\frac{-(\mu_j-\bar{\mu})^2}{2\Delta}}}{\sqrt{2\pi\Delta}}\right)\left(1+\alpha\sum_\ell \mu_\ell\right)\right] \approx \left(\frac{1}{1-\alpha^2 k\bar{V}}\right)[1+\alpha k\bar{\mu}]\ . \tag{38}$$

Computing σ_k^2 from Eq. (30) therefore leads after multiple cancellations to the final result

$$\sigma_k^2 \approx \left(\frac{1}{1-\alpha^2 k\bar{V}}\right)^2 \Delta\alpha^2 k\ , \tag{39}$$

where $\bar{V}$ and Δ are of course solutions of the system derived earlier on.

Eq. (39) indeed predicts an approximately linear behavior of σ_k^2 with k,

$$\sigma_k^2 \approx \xi(\alpha,c)k\ , \tag{40}$$

which is consistent with Fig. 5 of the main text, and a slope $\xi(\alpha,c)$ that is given by the following formula after simplifications

$$\xi(\alpha,c) = \frac{2\alpha^4 c\left(8\alpha^2+4\alpha^2c^2-4\alpha\sqrt{1-4\alpha^2(c-1)}+c\left(-\sqrt{1-4\alpha^2(c-1)}+4\alpha\left(\sqrt{1-4\alpha^2(c-1)}-3\alpha\right)-1\right)+2\right)}{(c-1)(\alpha c-1)^2\left(4\alpha^2(c-1)-1\right)\left(-2\alpha^2c+\sqrt{1-4\alpha^2(c-1)}+1\right)^2}\ . \tag{41}$$

The slope is increasing in c for α fixed, and increasing in α for c fixed. It also matches reasonably well the numerical simulations in the region of the (α,c) parameter space that meets the various requirements for the approximation to hold (essentially, c not too small, and $\alpha \ll 1$).

In Fig. S1 below, we present a comparison between the pdf $P(K_s)$ of the shifted Katz centrality K_s obtained by randomly generated Erdős-Rényi networks using the inversion formula Eq. (5) of the main text, and the Dirac comb approximate formula Eq. (51) of the main text with $K = K_s - 1$. For the simulations, we use an ensemble of 30 Erdős-Rényi networks of size $N = 5000$ with $c = 30$ and $\alpha = 1/45$.

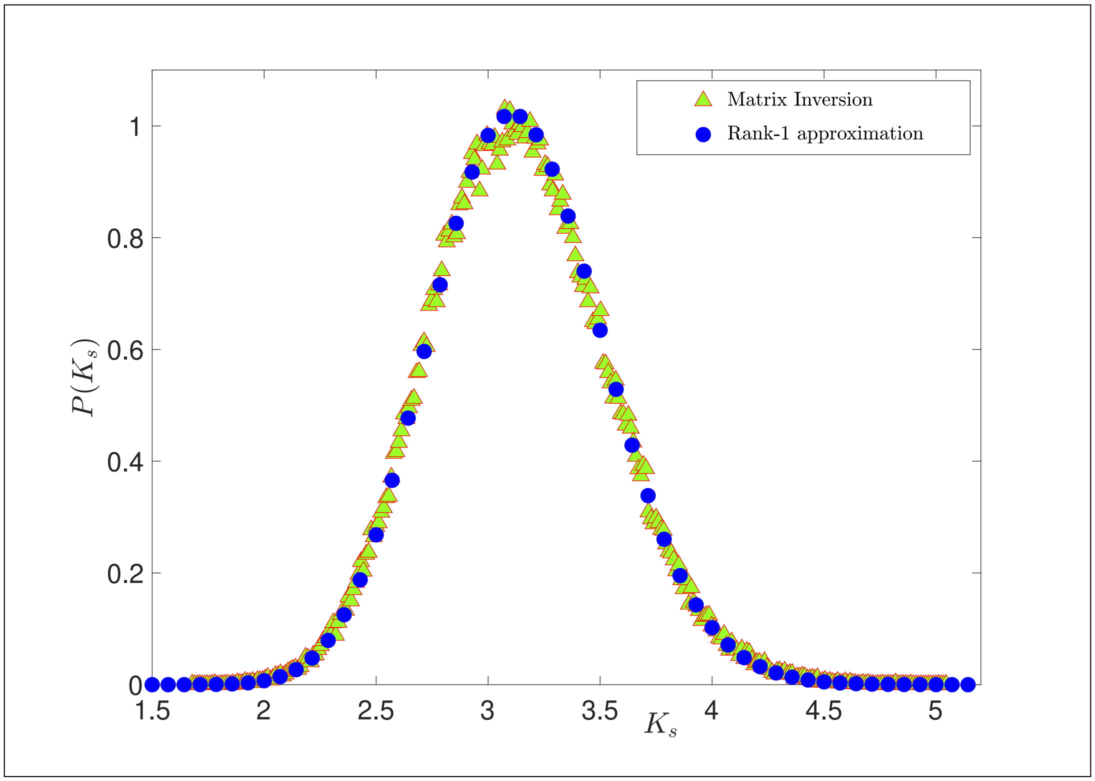

Fig. S1. Probability density function $P(K_s)$ of the shifted Katz centrality K_s for an ensemble of 30 Erdős-Rényi networks of size $N = 5000$ with $c = 30$ and $\alpha = 1/45$. Green triangles: histogram of node centralities from randomly generated E-R networks using the inversion formula Eq. (5) of the main text. Blue dots: Dirac comb approximate formula Eq. (51) of the main text with $K = K_s - 1$.

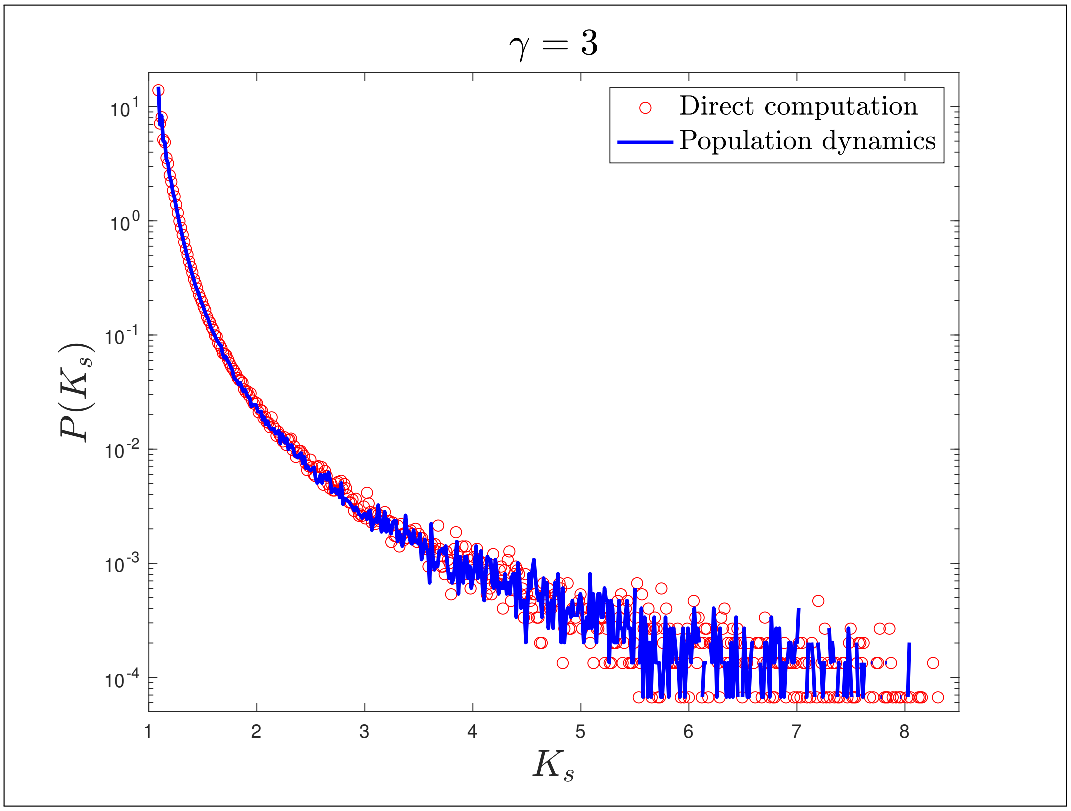

Fig. S2. Probability density function $P(K_s)$ in semi-logarithmic scale of the shifted Katz centrality with $\alpha = 1/40$ computed over an ensemble of 100 Scale Free graphs of size $N = 10000$ with parameter $\gamma = 3$ and minimum degree $k_{min} = 3$ by direct matrix inversion from Eq. (5) of the main text (red circles). Blue solid line: distribution of the population $\tilde{\mathbf{M}}$ after reaching equilibrium, with $N_P = 10^6$ population members and 100 updating sweeps (see Section 6 of the main text for details).

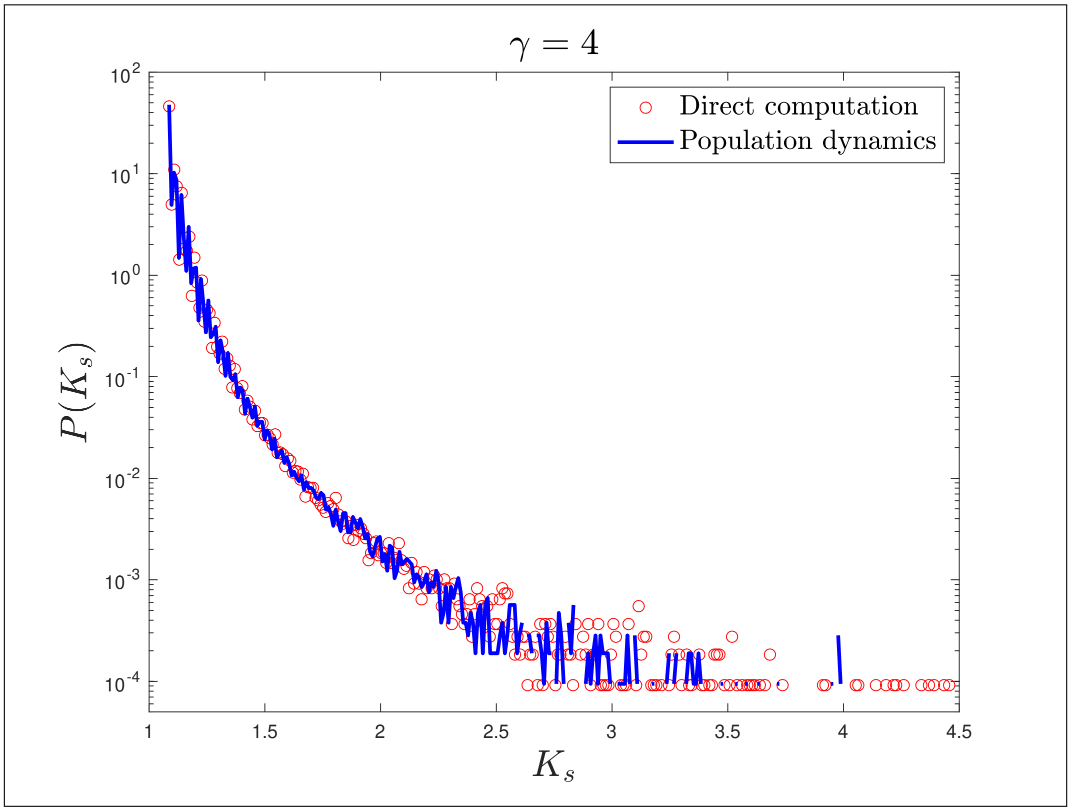

Fig. S3. Probability density function $P(K_s)$ in semi-logarithmic scale of the shifted Katz centrality with $\alpha = 1/40$ computed over an ensemble of 100 Scale Free graphs of size $N = 10000$ with parameter $\gamma = 4$ and minimum degree $k_{min} = 3$ by direct matrix inversion from Eq. (5) of the main text (red circles). Blue solid line: distribution of the population $\tilde{\mathbf{M}}$ after reaching equilibrium, with $N_P = 10^6$ population members and 100 updating sweeps (see Section 6 of the main text for details).

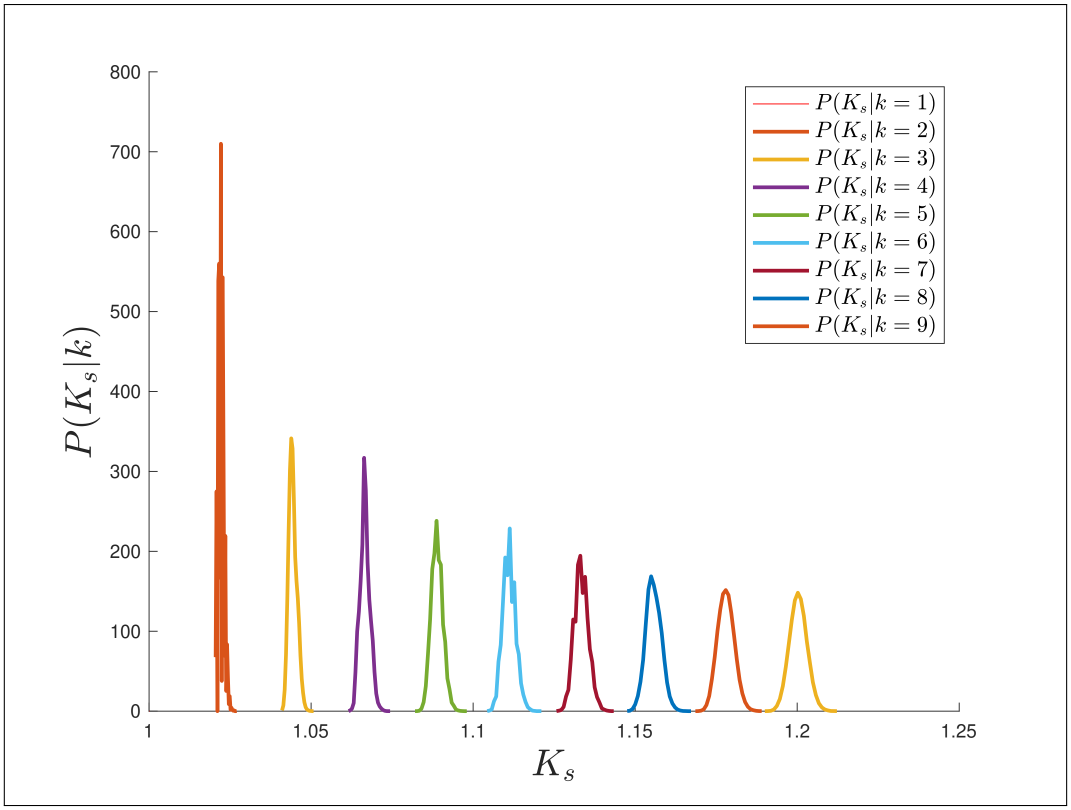

Fig. S4. Conditional pdf $P(K_s|k)$ of the shifted Katz centrality $K_s = K + 1$ of nodes of degree k for an ensemble of graphs with Poissonian degree distribution with mean $c = 4$, $\alpha = 1/40$ and population size $N_P = 10^5$ (see Eq. (38) of the main text). The curves are obtained via Monte Carlo sampling of the integral in Eq. (39) of the main text after the population has reached equilibrium. We display curves for degree up to $k = 9$.

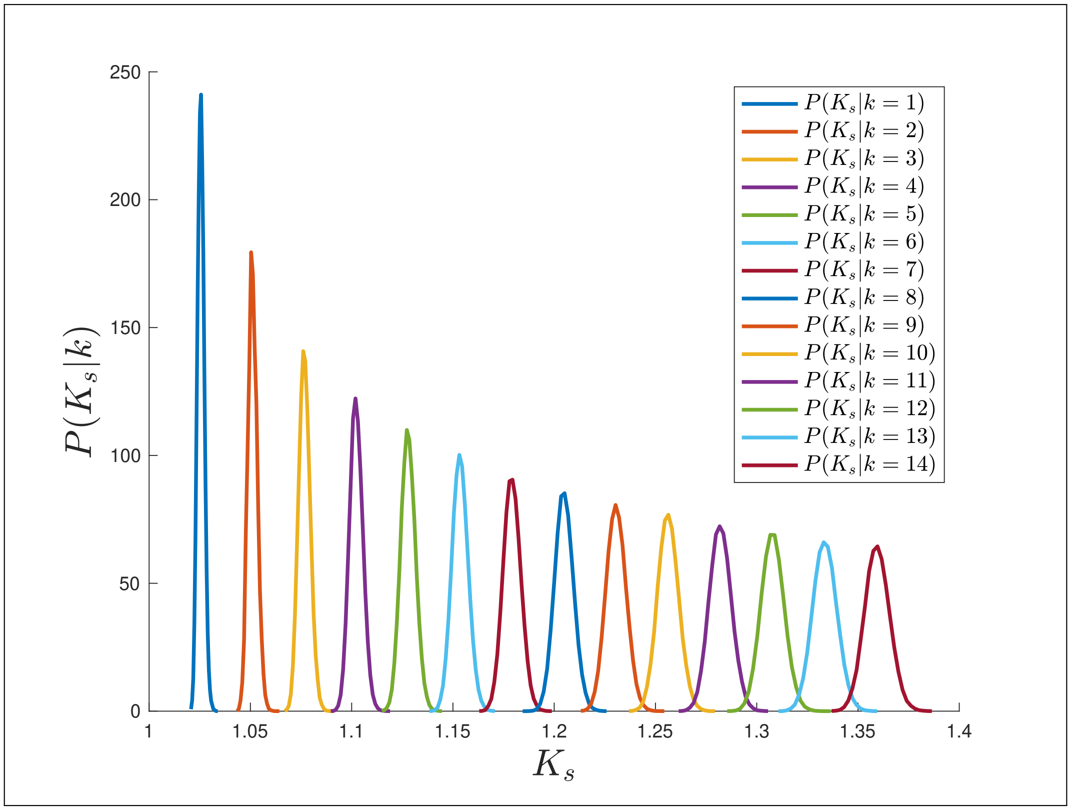

Fig. S5. Conditional pdf $P(K_s|k)$ of the shifted Katz centrality $K_s = K + 1$ of nodes of degree k for an ensemble of graphs with Poissonian degree distribution with mean $c = 10$, $\alpha = 1/40$ and population size $N_P = 10^5$ (see Eq. (38) of the main text). The curves are obtained via Monte Carlo sampling of the integral in Eq. (39) of the main text after the population has reached equilibrium. We display curves for degree up to $k = 14$.

C. Statistical validation on empirical networks - further experiments. In this subsection, we report further experiments using our statistical validation method via Katz centrality distributions. As a consistency check we first show that, indeed, if we generate an instance of a synthetic Erdős-Rényi network and we carry out the validation procedure on it, the Katz centrality of all nodes is compatible with the 'null' model as it should (see Fig. S6, S7). Next, we show two further validation experiments with empirical networks using:

- The Ego-Facebook dataset, comprising the matrix of interactions of 4039 users (nodes) and 176468 friendships (edges) (2). In fig. S8 we show the scatter plot of the Katz centrality versus the average centrality conditional on degree, highlighting the different classes of nodes at a given confidence level (over- and under- expressed with respect to the null model). In fig. S9 we display the values of empirical Katz centralities vs. the distribution of conditional probability of the null model for nodes of given degree ($k = 25$).

- The Arxiv HEP-TH (High Energy Physics - Theory) collaboration network from the e-print ArXiv covering scientific collaborations between authors in papers submitted to the High Energy Physics - Theory category (3). It contains 9877 nodes. If an author i co-authored a paper with author j, the graph contains an undirected edge connecting i to j. The data covers papers in the period from January 1993 to April 2003. In fig. S10, we plot the Katz centrality versus the average centrality conditional on degree, highlighting the different classes of nodes at a given confidence level (over- and under- expressed with respect to the null model) as well as the top 1% nodes ranked by Katz centrality.

Finally, in Table S1 we report the full list of airports considered in the example in the Main Text (see Fig. 10 - main text) with full details on airport locations as well as their classification according to our validation procedure (over-/under-expressed) and their degree class.

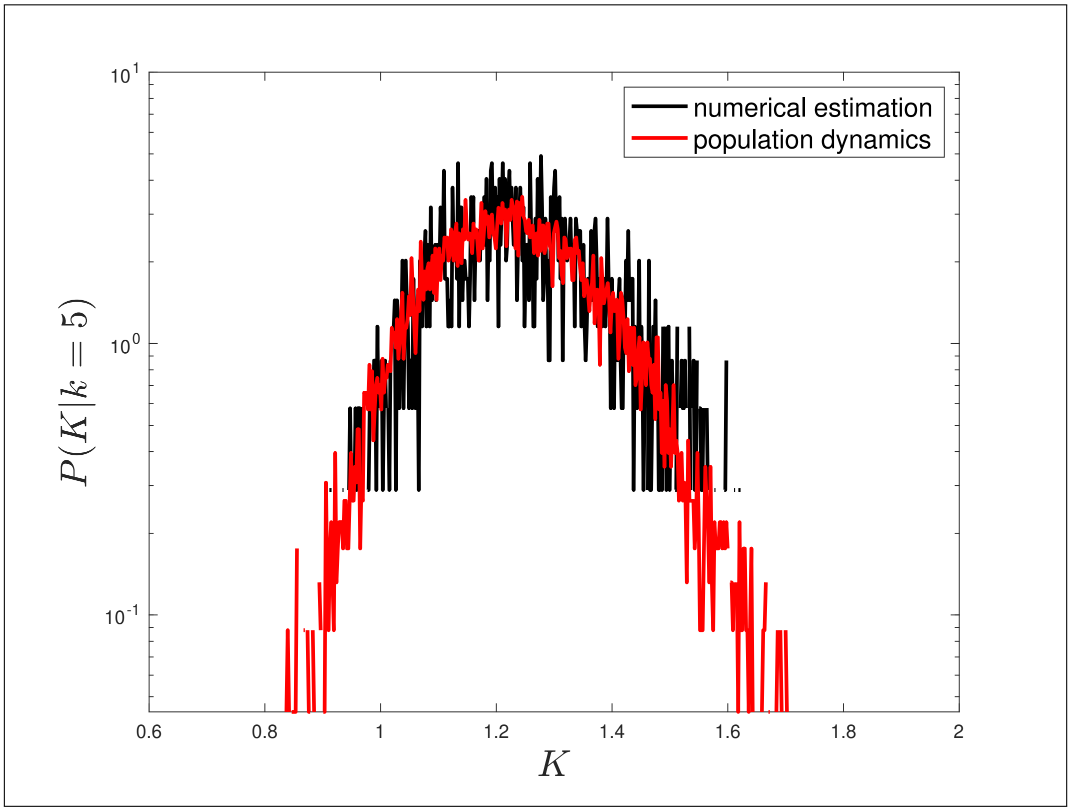

Fig. S6. Conditional distribution of Katz centrality for nodes of degree 5 in a single instance of an Erdős-Rényì network with 10000 nodes and an average degree of 5. The black line represents the probability distribution estimated from the actual Katz centralities calculated for the network nodes. The red line is derived from the stationary solution of population dynamics equations for a population of size 10000, using a Monte Carlo simulation of 100000 samples to estimate the integral in equation 39 of the main text.

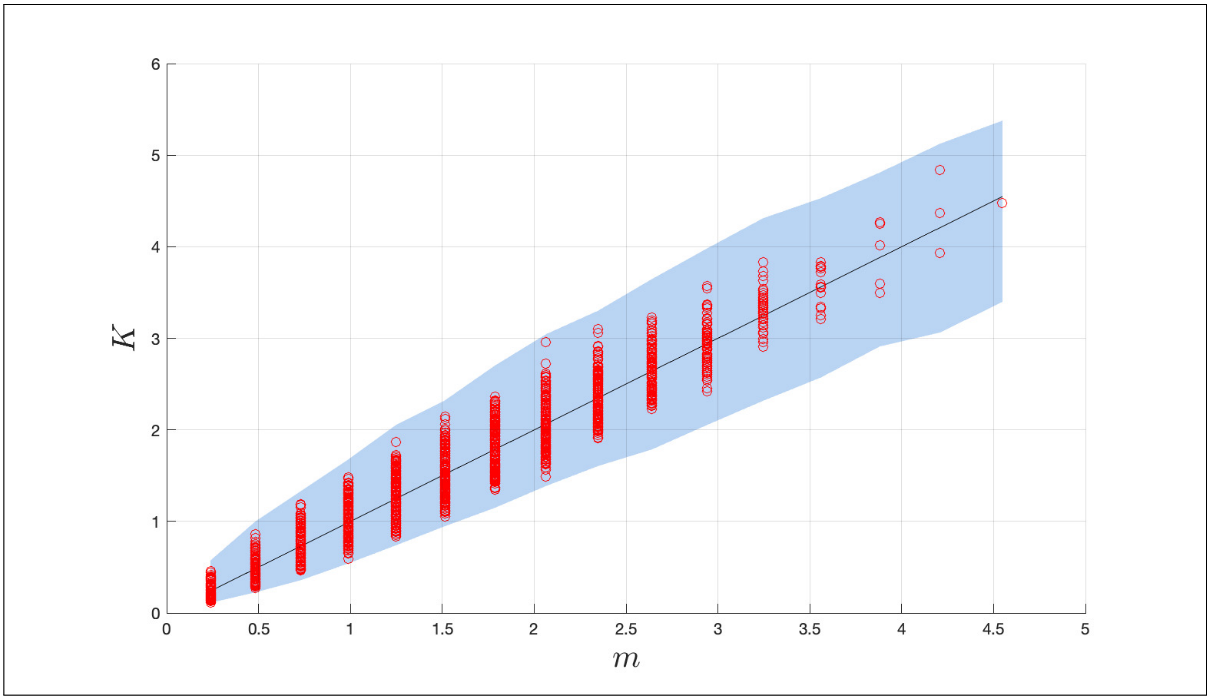

Fig. S7. Average centrality conditional on node's degree vs. Katz centrality. Each dot corresponds to a node of an Erdős-Rényi random network of size 10000 with average degree 5. The blue region corresponds to values within 99% confidence bounds as per the validation procedure discussed in section 7 of the main text. All nodes have a value of centrality compatible with that of the null model.

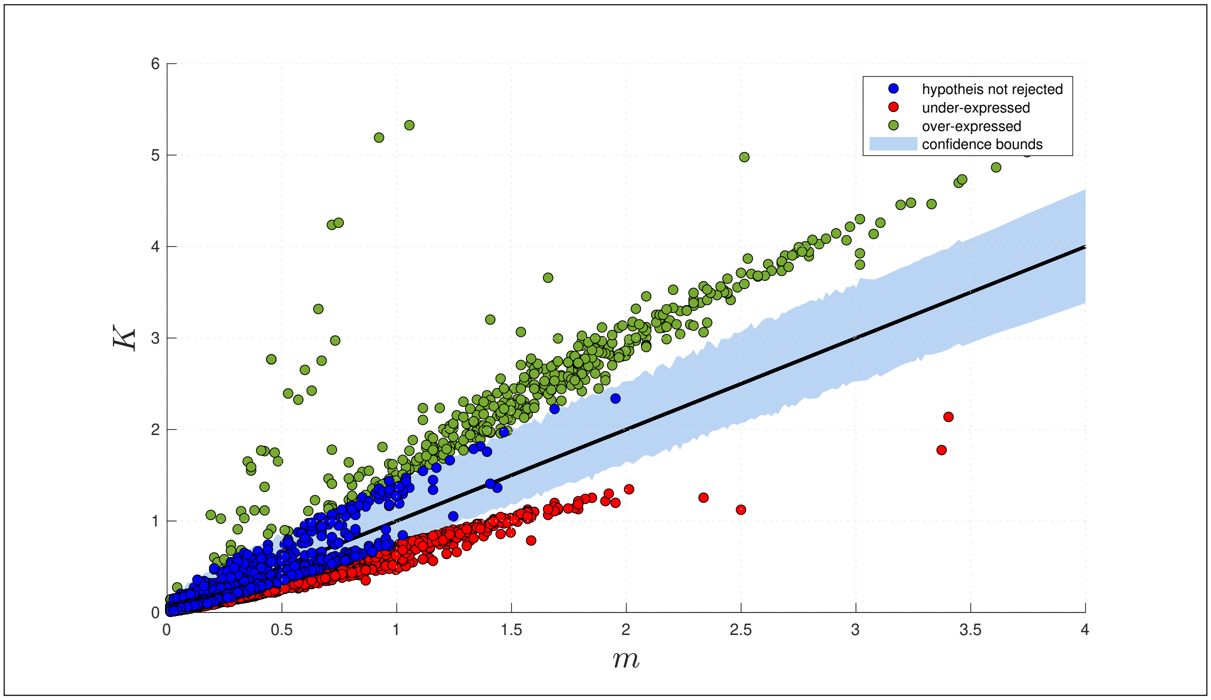

Fig. S8. Katz centrality (computed with $\alpha = 1/175$) vs. average centrality conditional on degree for the Ego-Facebook network (2). The shaded region represents 0.999 confidence bounds. Red circles correspond to nodes with under-expressed centrality. Green circles correspond to nodes with over-expressed centrality. Blue circles correspond to nodes with centrality within the confidence bounds. The black line corresponds to the diagonal.

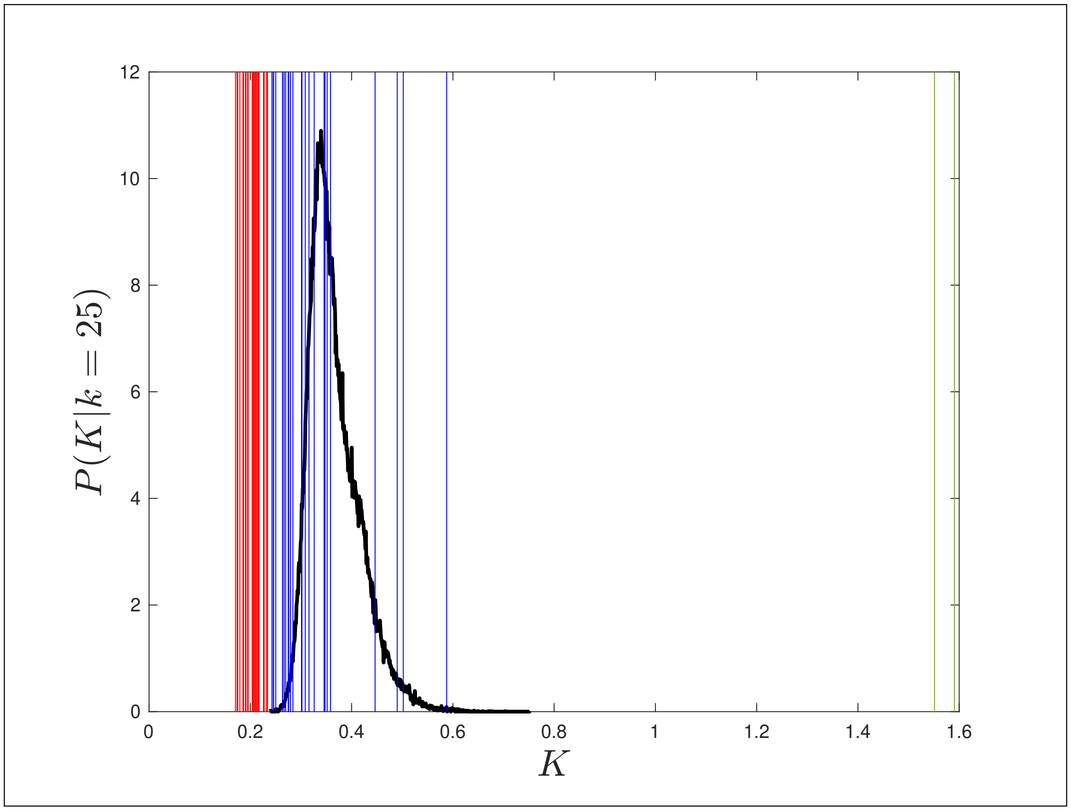

Fig. S9. Comparison between (black curve) the conditional distribution $P(K|k = 25)$ with $\alpha = 1/175$, obtained from the population dynamics (Eqs. 36-37-39) with $N_P = 10^4$ where the $p(k)$ is taken as the degree sequence of the empirical Ego-Facebook network (2), and (vertical lines) the different values taken by the centrality of nodes of degree $= 25$ in the Ego-Facebook dataset, comprising the matrix of interactions of 4039 users (nodes) and 176468 friendships (edges). Red lines correspond to nodes with under-expressed centrality. Green lines correspond to nodes with over-expressed centrality. Blue lines correspond to nodes with centrality within the confidence bounds.

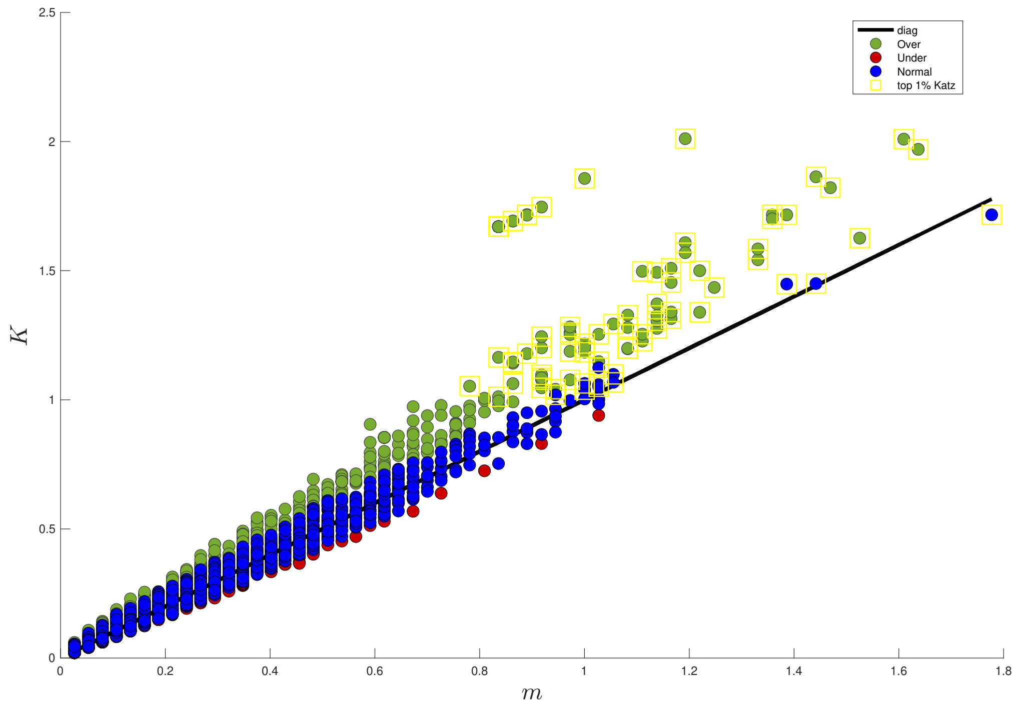

Fig. S10. Katz centrality (computed with $\alpha = 1/50$) vs. average centrality conditional on degree for the network (3) at 0.95 confidence level. Red circles correspond to nodes with under-expressed centrality. Green circles correspond to nodes with over-expressed centrality. Blue circles correspond to nodes with centrality within the confidence bounds. The black line corresponds to the diagonal. Yellow squares highlight top 1% nodes by centrality.

Table S1 Summary of nodes with labels (airport code and location) arranged by degree class [1−10], [11−50], [51−100], [100+]. We also indicate whether nodes are under- or over-expressed with respect to the random null model with the same degree sequence (see Sec. 7 and example in Fig. 10 in the Main Text.)

Katz	Degree	Airport Code	Location
**Degree [1-10]**			
Under	8	ADQ	Kodiak, Alaska, USA
	5	FYU	Fort Yukon, Alaska, USA
	3	TNK	Tununak, Alaska, USA
	7	YEV	Inuvik, Canada
	10	YFB	Iqaluit, Canada
	9	ANI	Aniak, Alaska, USA
	3	CKD	Crooked Creek, Alaska, USA
	6	GAL	Galena, Alaska, USA
	6	HSL	Huslia, Alaska, USA
	4	SLQ	Sleetmute, Alaska, USA
	1	SPB	San Luis, Colorado, USA
	1	SSB	San Luis, Colorado, USA
	3	BKY	Bukavu, DR Congo
	3	BUX	Bunia, DR Congo
	4	GOM	Goma, DR Congo
	9	HGU	Mount Hagen, Papua New Guinea
	9	LAE	Lae, Papua New Guinea
	7	MAG	Madang, Papua New Guinea
	8	RAB	Rabaul, Papua New Guinea
	10	AMQ	Ambon, Indonesia
	3	JJU	Qaqortoq, Greenland
Over	7	GWT	Westerland, Germany
	8	KLX	Kalamata, Greece
	8	SMI	Samos, Greece
	8	BDA	Bermuda
	6	INN	Innsbruck, Austria
	9	PVK	Preveza, Greece
	7	SPC	La Palma, Spain
	9	HOG	Holguin, Cuba
	5	VGO	Vigo, Spain
	8	ISU	Sulaymaniyah, Iraq
	10	OVD	Asturias, Spain
	5	ASR	Kayseri, Turkey
Normal	1	TGK	Taganrog, Russia
	1	KCK	Kirensk, Russia
	6	MJZ	Mirny, Russia
	1	ODO	Bodaybo, Russia
	1	UKX	Ust-Kut, Russia
	2	ULK	Lensk, Russia
	7	TKU	Turku, Finland
	4	VBY	Visby, Sweden
	6	VXO	Vaxjo, Sweden
	2	AOS	Amook Bay, Alaska, USA
	2	KKB	Kitoi Bay, Alaska, USA
	2	KLN	Larsen Bay, Alaska, USA
	2	KOZ	Ouzinkie, Alaska, USA
	2	SYB	Seal Bay, Alaska, USA
	1	IQT	Iquitos, Peru
	5	CUZ	Cusco, Peru
	3	PCL	Pucallpa, Peru
	2	PEM	Puerto Maldonado, Peru
	3	TPP	Tarapoto, Peru
	2	OCC	Francisco de Orellana, Ecuador
	1	SCY	San Cristobal, Ecuador
	4	GEV	Gallivare, Sweden
	4	HAD	Halmstad, Sweden

Katz	Degree	Airport Code	Location
	2	JKG	Jonkoping, Sweden
	2	KOK	Kokkola, Finland
	2	KRF	Kramfors, Sweden
	2	KSD	Karlstad, Sweden
	2	LYC	Lycksele, Sweden
	3	MHQ	Mariehamn, Finland
	1	OER	Ornskoldsvik, Sweden
	3	POR	Pori, Finland
	7	TKU	Turku, Finland
	4	VBY	Visby, Sweden
	2	VHM	Vilhelmina, Sweden
	6	VXO	Vaxjo, Sweden
	2	AOS	Amook Bay, Alaska, USA
	2	KKB	Kitoi Bay, Alaska, USA
	2	KLN	Larsen Bay, Alaska, USA
	2	KOZ	Ouzinkie, Alaska, USA
	2	SYB	Seal Bay, Alaska, USA
	1	BOY	Bobo Dioulasso, Burkina Faso
	9	NIM	Niamey, Niger
	8	GES	General Santos, Philippines
	5	KLO	Kalibo, Philippines
	6	LGP	Legazpi, Philippines
	8	MPH	Caticlan, Philippines
	2	OZC	Ozamis, Philippines
	7	PPS	Puerto Princesa, Philippines
	2	RXS	Roxas City, Philippines
	1	SUG	Surigao, Philippines
	2	TUG	Tuguegarao, Philippines
	3	ZAM	Zamboanga, Philippines
**Degree [11-50]**			
**Under**	45	BSB	Brasília, Brazil
	18	CGB	Cuiabá, Brazil
	12	PLU	Belo Horizonte, Brazil
	15	KTN	Ketchikan, USA
	11	YWK	Wabush, Canada
	11	YZV	Sept-Îles, Canada
	18	YZF	Yellowknife, Canada
	32	TIJ	Tijuana, Mexico
	24	FAI	Fairbanks, USA
	29	BET	Bethel, USA
	29	CEB	Cebu, Philippines
	44	AEP	Buenos Aires, Argentina
	24	CWB	Curitiba, Brazil
	29	POA	Porto Alegre, Brazil
	11	YRT	Rankin Inlet, Canada
	34	ANC	Anchorage, USA
	16	OME	Nome, USA
	14	OTZ	Kotzebue, USA
	15	PAC	Panama City, Panama
	14	PMV	Porlamar, Venezuela
	17	LPB	La Paz, Bolivia
	50	AKL	Auckland, New Zealand
	21	CHC	Christchurch, New Zealand
	35	CNF	Belo Horizonte, Brazil
	30	CNS	Cairns, Australia
	22	ITM	Osaka, Japan
	16	JNU	Juneau, USA
	24	NAN	Nadi, Fiji
	32	PPT	Papeete, French Polynesia
	11	YYR	Goose Bay, Canada

Katz	Degree	Airport Code	Location
	19	BEL	Belém, Brazil
	26	CGH	São Paulo, Brazil
	18	SDU	Rio de Janeiro, Brazil
	48	DMK	Bangkok, Thailand
	20	MYY	Miri, Malaysia
	26	UPG	Makassar, Indonesia
	11	WIL	Nairobi, Kenya
	40	MHD	Mashhad, Iran
	38	THR	Tehran, Iran
	35	POM	Port Moresby, Papua New Guinea
	21	WLG	Wellington, New Zealand
	50	SFB	Sanford, USA
	21	HIR	Honiara, Solomon Islands
	12	SUV	Suva, Fiji
	21	VLI	Port Vila, Vanuatu
	17	BTH	Batam, Indonesia
	32	AZA	Phoenix, USA
	18	PGD	Punta Gorda, USA
	31	PIE	St. Petersburg, USA
	18	YXL	Sioux Lookout, Canada
	40	NAY	Beijing, China
Over	39	GYD	Baku, Azerbaijan
	49	BOD	Bordeaux, France
	18	DRS	Dresden, Germany
	11	GRZ	Graz, Austria
	33	OLB	Olbia, Italy
	21	SZG	Salzburg, Austria
	26	JAX	Jacksonville, USA
	12	NDR	Nador, Morocco
	47	TLS	Toulouse, France
	12	TNG	Tangier, Morocco
	48	PUJ	Punta Cana, Dominican Republic
	26	HRG	Hurghada, Egypt
	17	PRN	Pristina, Kosovo
	49	BGO	Bergen, Norway
	23	BIA	Bastia, France
	35	BIO	Bilbao, Spain
	43	BRE	Bremen, Germany
	41	BRI	Bari, Italy
	39	CFU	Corfu, Greece
	50	CTA	Catania, Italy
	21	CWL	Cardiff, UK
	42	DBV	Dubrovnik, Croatia
	11	JMK	Mykonos, Greece
	11	JTR	Santorini, Greece
	30	KGS	Kos, Greece
	28	LEJ	Leipzig, Germany
	33	LIN	Milan Linate, Italy
	32	NUE	Nuremberg, Germany
	39	PMO	Palermo, Italy
	19	POZ	Poznan, Poland
	14	PUY	Pula, Croatia
	49	RAK	Marrakech, Morocco
	37	SPU	Split, Croatia
	22	SUF	Lamezia Terme, Italy
	29	TRN	Turin, Italy
	23	VRN	Verona, Italy
	31	WRO	Wroclaw, Poland
	16	XRY	Jerez, Spain
	28	ZAG	Zagreb, Croatia

Katz	Degree	Airport Code	Location
	25	TBS	Tbilisi, Georgia
	44	IKA	Tehran, Iran
	45	ADB	Izmir, Turkey
	21	BJV	Bodrum, Turkey
	29	DLM	Dalaman, Turkey
	12	ODS	Odessa, Ukraine
	47	CVG	Cincinnati, USA
	14	GSO	Greensboro, USA
	39	MSY	New Orleans, USA
	16	SYR	Syracuse, USA
	14	XNA	Bentonville, USA
	25	KIV	Chisinau, Moldova
	39	CHQ	Chania, Greece
	41	SOF	Sofia, Bulgaria
	18	TIA	Tirana, Albania
	13	ZTH	Zakynthos, Greece
	26	FLR	Florence, Italy
	32	GOT	Gothenburg, Sweden
	26	SXB	Strasbourg, France
	42	AUS	Austin, USA
	26	BDL	Hartford, USA
	20	BUF	Buffalo, USA
	20	CHS	Charleston, USA
	33	CMH	Columbus, USA
	11	COS	Colorado Springs, USA
	16	DAY	Dayton, USA
	35	IND	Indianapolis, USA
	34	LHE	Lahore, Pakistan
	29	MBJ	Montego Bay, Jamaica
	43	MCI	Kansas City, USA
	30	MKE	Milwaukee, USA
	49	NCL	Newcastle, UK
	20	OMA	Omaha, USA
	19	ORF	Norfolk, USA
	37	PIT	Pittsburgh, USA
	16	POP	Puerto Plata, Dominican Republic
	39	RDU	Raleigh, USA
	19	RIC	Richmond, USA
	18	ROC	Rochester, USA
	30	RTM	Rotterdam, Netherlands
	13	SAV	Savannah, USA
	23	SDF	Louisville, USA
	37	SVG	Stavanger, Norway
	25	DJE	Djerba, Tunisia
	30	FNC	Funchal, Portugal
	47	GDN	Gdansk, Poland
	30	JER	Jersey, UK
	36	LCY	London City, UK
	15	LEI	Almeria, Spain
	26	MAH	Menorca, Spain
	28	NBE	Enfidha, Tunisia
	38	SVQ	Seville, Spain
	46	VNO	Vilnius, Lithuania
	45	BEY	Beirut, Lebanon
	39	BLL	Billund, Denmark
	17	GOA	Genoa, Italy
	22	LJU	Ljubljana, Slovenia
	27	MRU	Mauritius
	22	AGA	Agadir, Morocco
	34	KEF	Keflavik, Iceland

Katz	Degree	Airport Code	Location
	41	MSQ	Minsk, Belarus
	28	TLL	Tallinn, Estonia
	13	AAL	Aalborg, Denmark
	12	TGD	Podgorica, Montenegro
	12	PWM	Portland, USA
	25	PFO	Paphos, Cyprus
	30	ORK	Cork, Ireland
	22	SCQ	Santiago de Compostela, Spain
	14	SEN	Southend, UK
	26	SNN	Shannon, Ireland
	20	EBL	Erbil, Iraq
	11	RZE	Rzeszow, Poland
	16	SID	Sal, Cape Verde
	13	BVC	Boa Vista, Cape Verde
	29	BFS	Belfast, UK
	16	LWO	Lviv, Ukraine
	12	BRN	Bern, Switzerland
Normal	18	AER	Sochi, Russia
	14	CEK	Chelyabinsk, Russia
	21	KZN	Kazan, Russia
	16	MRV	Mineralnye Vody, Russia
	28	IKT	Irkutsk, Russia
	23	YKS	Yakutsk, Russia
	21	ABJ	Abidjan, Ivory Coast
	30	ACC	Accra, Ghana
	16	BKO	Bamako, Mali
	20	COO	Cotonou, Benin
	32	DKR	Dakar, Senegal
	16	LFW	Lomé, Togo
	15	OUA	Ouagadougou, Burkina Faso
	18	CLO	Cali, Colombia
	19	GYE	Guayaquil, Ecuador
	26	UIO	Quito, Ecuador
	19	BDS	Brindisi, Italy
	30	TRF	Sandefjord, Norway
	41	DWC	Dubai World Central, UAE
	50	GIG	Rio de Janeiro, Brazil
	11	GYN	Goiânia, Brazil
	12	RAO	Ribeirão Preto, Brazil
	30	SSA	Salvador, Brazil
	21	DLA	Douala, Cameroon
	12	FNA	Freetown, Sierra Leone
	44	LOS	Lagos, Nigeria
	20	YQB	Quebec City, Canada
	13	JIB	Djibouti, Djibouti
	20	DRW	Darwin, Australia
	38	HAK	Haikou, China
	35	IND	Indianapolis, USA
	34	LHE	Lahore, Pakistan
	29	MBJ	Montego Bay, Jamaica
	43	MCI	Kansas City, USA
	30	MKE	Milwaukee, USA
	49	NCL	Newcastle, UK
	20	OMA	Omaha, USA
	19	ORF	Norfolk, USA
	37	PIT	Pittsburgh, USA
	16	POP	Puerto Plata, Dominican Republic
	39	RDU	Raleigh, USA
	19	RIC	Richmond, USA
	18	ROC	Rochester, USA

Katz	Degree	Airport Code	Location
	30	RTM	Rotterdam, Netherlands
**Degree [51-100]**			
Under	78	MNL	Manila, Philippines
	89	CKG	Chongqing, China
	70	CSX	Changsha, China
	59	DLC	Dalian, China
	58	HRB	Harbin, China
	95	KMG	Kunming, China
	85	SYD	Sydney, Australia
	86	SZX	Shenzhen, China
	86	XIY	Xi'an, China
	80	SHJ	Sharjah, UAE
	69	SHA	Shanghai, China
	61	BNE	Brisbane, Australia
	51	VCP	Campinas, Brazil
Over	73	BRS	Bristol, UK
	55	LCA	Larnaca, Cyprus
	73	BRS	Bristol, UK
	55	LCA	Larnaca, Cyprus
	83	LPA	Las Palmas, Spain
	85	TFS	Tenerife South, Spain
	96	YUL	Montreal, Canada
	87	HAM	Hamburg, Germany
	51	NAP	Naples, Italy
	64	BLQ	Bologna, Italy
	93	CGN	Cologne, Germany
	93	CMN	Casablanca, Morocco
	78	LYS	Lyon, France
	82	CAI	Cairo, Egypt
	79	TLV	Tel Aviv, Israel
	81	CUN	Cancun, Mexico
	65	AYT	Antalya, Turkey
	66	BSL	Basel, Switzerland
	52	BEG	Belgrade, Serbia
	81	BHX	Birmingham, UK
	77	BUD	Budapest, Hungary
	89	EDI	Edinburgh, UK
	64	FAO	Faro, Portugal
	51	HAJ	Hanover, Germany
	89	HEL	Helsinki, Finland
	62	HER	Heraklion, Greece
	59	IBZ	Ibiza, Spain
	52	KRK	Krakow, Poland
	90	NCE	Nice, France
	94	PRG	Prague, Czech Republic
	65	PSA	Pisa, Italy
	51	RHO	Rhodes, Greece
	55	SKG	Thessaloniki, Greece
	81	STR	Stuttgart, Germany
	61	TUN	Tunis, Tunisia
	73	VCE	Venice, Italy
	78	WAW	Warsaw, Poland
	69	SXF	Berlin Schönefeld, Germany
	68	RIX	Riga, Latvia
	58	AMM	Amman, Jordan
	62	KBP	Kyiv, Ukraine
	68	OTP	Bucharest, Romania
	90	AUH	Abu Dhabi, UAE
	86	MRS	Marseille, France
	56	NTE	Nantes, France

Katz	Degree	Airport Code	Location
	63	GLA	Glasgow, UK
	51	LBA	Leeds, UK
	56	LUX	Luxembourg
	57	ACE	Lanzarote, Spain
	96	ALC	Alicante, Spain
	55	FUE	Fuerteventura, Spain
	77	MLA	Malta
	57	VLC	Valencia, Spain
	61	OPO	Porto, Portugal
Normal	53	OVB	Novosibirsk, Russia
	67	SVX	Yekaterinburg, Russia
	57	LIM	Lima, Peru
	74	BOG	Bogota, Colombia
	92	GRU	São Paulo, Brazil
	61	STL	St. Louis, USA
	67	NBO	Nairobi, Kenya
	72	HGH	Hangzhou, China
**Degree [101-248]**			
Under	190	DME	Moscow, Russia
	217	ATL	Atlanta, USA
	150	CAN	Guangzhou, China
	108	CTU	Chengdu, China
	188	DFW	Dallas/Fort Worth, USA
	170	DEN	Denver, USA
Over	101	GVA	Geneva, Switzerland
	137	ZRH	Zurich, Switzerland
	124	ARN	Stockholm, Sweden
	105	OSL	Oslo, Norway
	163	BCN	Barcelona, Spain
	165	LGW	London Gatwick, UK
	147	DUS	Dusseldorf, Germany
	192	MUC	Munich, Germany
	110	TXL	Berlin Tegel, Germany
	248	AMS	Amsterdam, Netherlands
	153	BRU	Brussels, Belgium
	162	JFK	New York John F. Kennedy, USA
	117	AGP	Malaga, Spain
	104	ATH	Athens, Greece
	240	CDG	Paris Charles de Gaulle, France
	144	DUB	Dublin, Ireland
	162	FCO	Rome, Italy
	244	FRA	Frankfurt, Germany
	172	LHR	London Heathrow, UK
	103	LIS	Lisbon, Portugal
	159	MAD	Madrid, Spain
	148	MAN	Manchester, UK
	113	MXP	Milan Malpensa, Italy
	139	VIE	Vienna, Austria
	104	NRT	Tokyo Narita, Japan
	144	SVO	Moscow Sheremetyevo, Russia
	122	CPH	Copenhagen, Denmark
	118	DOH	Doha, Qatar
	153	EWR	Newark, USA
	147	YYZ	Toronto, Canada
	127	IAD	Washington Dulles, USA
	105	SFO	San Francisco, USA
Normal	113	LED	St. Petersburg, Russia
	206	ORD	Chicago O'Hare, USA
	122	BKK	Bangkok, Thailand

References

1. M. Paton, K. Akartunali, and D. J. Higham, *Centrality analysis for modified lattices*, SIAM J. Matrix Anal. Appl. **38**(3), 1055 (2017).
2. J. McAuley and J. Leskovec, *Learning to Discover Social Circles in Ego Networks*, NIPS'12: Proceedings of the 25th International Conference on Neural Information Processing Systems **1**, 539-547 [https://snap.stanford.edu/data/ego-Facebook.html] (2012).
3. J. Leskovec, J. Kleinberg, and C. Faloutsos, *Graph evolution: Densification and shrinking diameters*. ACM transactions on Knowledge Discovery from Data (TKDD) **1(1)** (2007): 2-es. [https://snap.stanford.edu/data/ca-HepTh.html]
